# Collective Behavior of Urease pH Clocks in Nano- and Microvesicles Controlled by Fast Ammonia Transport

**DOI:** 10.1021/acs.jpclett.2c00069

**Published:** 2022-02-21

**Authors:** Ylenia Miele, Stephen J. Jones, Federico Rossi, Paul A. Beales, Annette F. Taylor

**Affiliations:** † Department of Chemistry and Biology, University of Salerno, Via Giovanni Paolo II 132, 84084 Fisciano, Salerno, Italy; ‡ School of Chemistry and Astbury Centre for Structural Molecular Biology, University of Leeds, Leeds LS2 9JT, U.K.; § Department of Earth, Environmental and Physical Sciences, University of Siena, Pian dei Mantellini 44, 53100 Siena, Italy; ∥ Chemical and Biological Engineering, University of Sheffield, Sheffield S1 3JD, U.K.

## Abstract

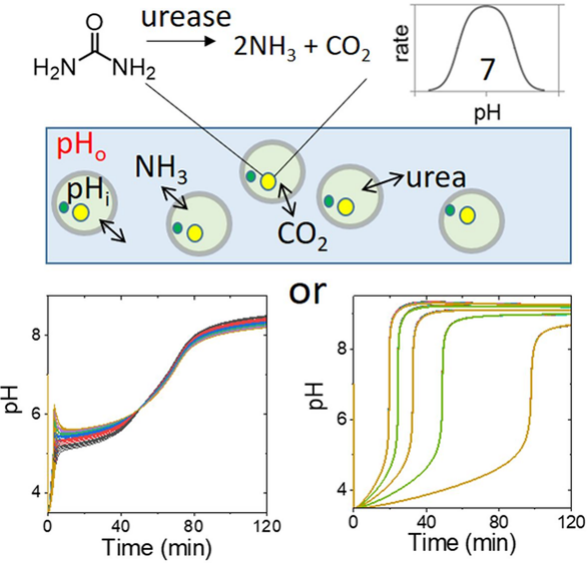

The transmission of chemical signals via an extracellular solution plays a vital role in collective behavior in cellular biological systems and may be exploited in applications of lipid vesicles such as drug delivery. Here, we investigated chemical communication in synthetic micro- and nanovesicles containing urease in a solution of urea and acid. We combined experiments with simulations to demonstrate that the fast transport of ammonia to the external solution governs the pH–time profile and synchronizes the timing of the pH clock reaction in a heterogeneous population of vesicles. This study shows how the rate of production and emission of a small basic product controls pH changes in active vesicles with a distribution of sizes and enzyme amounts, which may be useful in bioreactor or healthcare applications.

Lipid vesicles, or liposomes, are employed in enzyme bioreactors^1,2^ and healthcare applications^3^ and are also used for the construction of artificial cells with bioinspired dynamics.^4−6^ The lipid membranes provide a protective layer with reduced permeability to large molecules and ionic species, and the release of chemicals from the vesicles can be used for molecular communication.^7−9^ Here, we investigated the role of the emission of base on the urease reaction when the enzyme was confined in synthetic nano- or microvesicles. Urease catalyzes the hydrolysis of urea, producing ammonia.^10^ In aqueous-phase experiments the reaction displays pH-dependent feedback and a rapid switch, referred to as a pH clock, after an induction period where the pH increases slowly.^11^ The reaction is widely used in materials and sensing applications,^12−17^ and urease has been encapsulated in vesicles and polymerosomes;^18−22^ however, the influence (if any) of compartmentalization and chemical communication on the process is not well understood.

Collective behavior has been mainly investigated with the inorganic Belousov–Zhabotinsky oscillating reaction in emulsion microdroplets or particles and vesicles.^23−26^ The period of the reaction depended on the catalyst loading and the particle size, and the products diffused between compartments, synchronizing the oscillations or driving more complex responses.^27−31^ In the encapsulation of more biologically relevant DNA and RNA transcriptional oscillators^32^ and protein oscillators,^33^ all the reactive species were confined to the microdroplets or vesicles; however, with urease-encapsulated vesicles, neutral acidic (CO_2_) and basic products (NH_3_) can diffuse into the surrounding solution. The methods for producing vesicles typically result in a distribution of sizes and enzyme content and so a variation in the pH clock time in individual vesicles might be expected in the absence of a collective response.^34,35^ Theoretical work also suggested that autonomous pH oscillations may occur in urease vesicles providing there is the sufficiently fast transport of acid from the external solution (*P*_H+_ > 10^–5^ m s^–1^); to date, however, these have not been observed in experiments.^36−38^ We show that the fast transport of ammonia controls the pH–time profile and synchronizes the pH change in the vesicles; here, the term synchronization is used to refer to a change in behavior (low to high pH) occurring at the same time in a heterogeneous population.

Nanovesicles were prepared using phospholipid film hydration and extrusion and encapsulated a solution of urease, pyranine, and HCl (SI 1.2–1.3 and Figure 1a). As the urease (Sigma-Aldrich type III) is not pure, we report enzyme concentrations in units per milliliter rather than micromoles. The reaction was initiated by adding a urea/HCl solution to a vesicle solution in a microcuvette. The ratio of the absorbance of pyranine at 450 and 405 nm was used to estimate the (apparent) pH using a calibration curve with a fitted theoretical relation (SI 1.4 and Figure S1),^39,40^ and the total number of vesicles in the 500 μL sample was on the order of *N* ∼ 10^11^ (SI 1.5).

**Figure 1:**
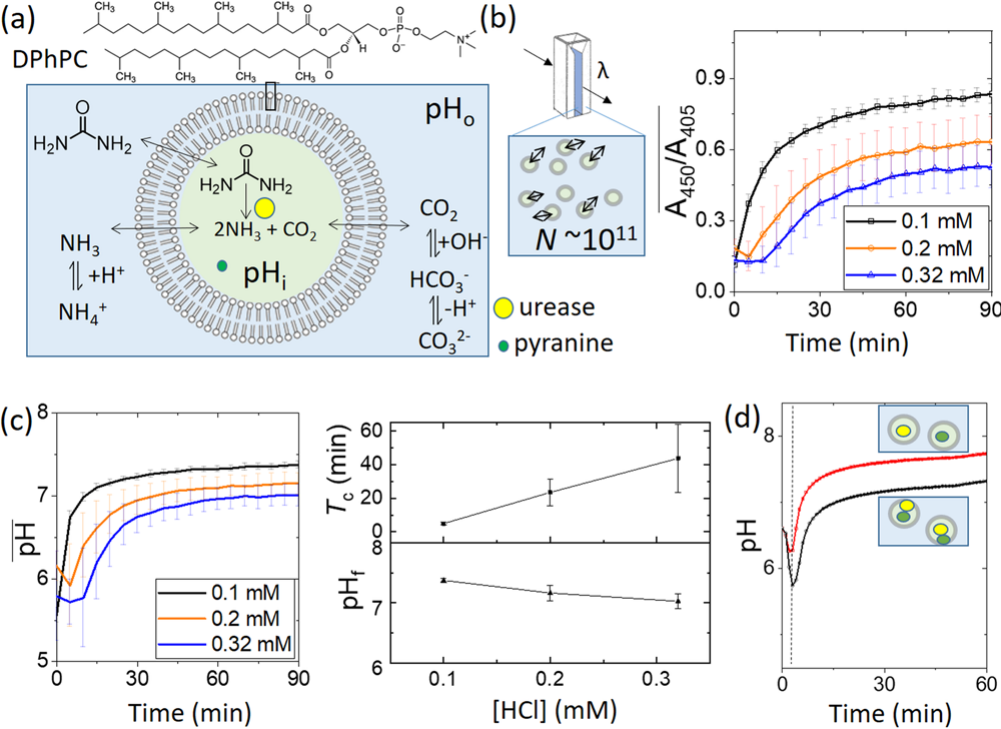
Urease pH clock reaction in nanovesicles of diameter *D* ∼ 200 nm that were prepared using 1,2-diphytanoyl-*sn*-glycero-3-phosphocholine (DPhPC), urease (220 U mL^–1^), HCl (0.1–0.32 mM), and pyranine (20 mM) and placed in a solution of urea (50 mM) and HCl (0.1–0.32 mM). (a) Schematic of the reaction with urease and pyranine confined to the vesicle (pH*_i_*), with urea in the outer solution (pH_o_) and the relevant equilibria. (b) Average ratio of absorbance from vesicles in a microcuvette in a urea/acid solution and the estimated total number of vesicles, *N*, in 500 μL. (c) The average pH in time obtained from the ratio of the absorbance in panel b, showing the average clock time *T*_c_ (here to pH 6.75) and the final pH as a function of initial acid concentration. (d) Comparison of the pH in time in experiments with [HCl] = 0.2 mM where pyranine was included in the same vesicles as the enzyme (lower black curve) and where pyranine and the enzyme were in separate vesicles (upper red curve). The dotted vertical line corresponds to mixing time. Error bars indicate the standard error from three independent experiments.

The ratio, *A*_450_/*A*_405_, is shown in time for different initial acid concentrations in Figure 1b, and the corresponding pH is in Figure 1c. In the aqueous phase, the clock time, *T*_c_, was defined as the time to reach pH 7, where the urease reaction rate was at a maximum, and depended on the acid concentration, the urea, and the amount of enzyme present in solution.^11^ The switch in pH was generally less sharp in the nanovesicles compared to that of the pH clocks in the aqueous phase, with a higher initial pH (after mixing) and a lower final pH (SI 1.6). However, the average clock time increased with the initial acid concentration as expected (Figure 1c).

Evidence for the increase in the amount of ammonia in the outer solution was obtained through experiments in which two populations of vesicles were prepared, one with pyranine but no enzyme and one with enzyme but no pyranine. When the two were mixed and urea solution was added, the pH increased, demonstrating the transfer of ammonia to the urease-free vesicles via the outer solution (Figure 1d). To rule out the possibility that enzymes from burst vesicles could contribute to the overall pH change in the cuvette, Triton-X was added to the solution to rupture all the vesicles. No increase in absorbance was observed in time (Figure S3).

The slow increase in pH in the nanovesicles obtained using absorbance measurements may arise from the average of a broad distribution in clock times in a diverse population or the increase may be slow and synchronized in all vesicles. In order to monitor the pH clock in individual vesicles, the urease reaction was performed in microvesicles prepared by the droplet transfer method (SI 2.1–2.2).^19,41,42^ A sample of vesicles was added to a reaction chamber, and the ratio of the fluorescence intensity (*F*_458_/*F*_405_) obtained using confocal microscopy was used to determine the (apparent) pH, with a fitted theoretical relationship (SI 2.3–2.4). The sizes of the vesicles ranged from 2–40 μm (Figure 2a), and the total number of vesicles in the chamber was *N* ∼ 10^4^ (SI 2.5).

**Figure 2:**
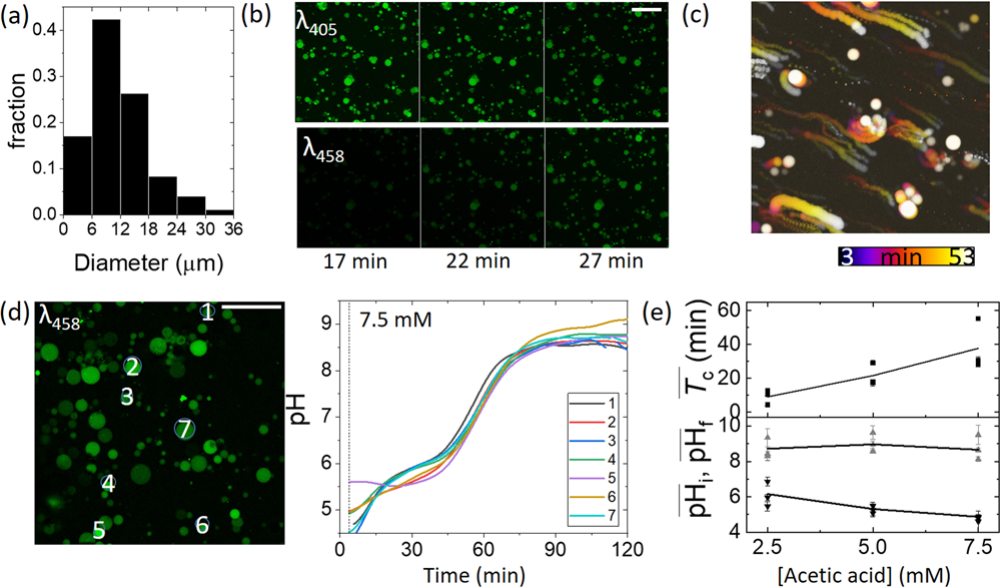
Urease pH clock reaction in synthetic microvesicles prepared using 1-palmitoyl-2-oleoyl-*sn*-glycero-3-phosphocholine (POPC), urease (80 U mL^–1^), pyranine (50 μM), and acetic acid (2.5–7.5 mM) in a solution of urea (32 mM) and acetic acid (2.5–7.5 mM). (a) Fraction of vesicles with a given diameter taken from reaction data with nine runs. (b) Confocal images in time of the urease pH clock reaction in vesicles with 5 mM acetic acid for two different excitation wavelengths. (c) Image overlay (λ_458nm_) for the entire time series in an experiment with an acid concentration of 2.5 mM showing vesicle motion. The colored bar indicates time. (d) Confocal image of the seven vesicles used in data analysis for 7.5 mM acid and the pH in time in each vesicle, where the dotted vertical line corresponds to the mixing time. (e) Clock time *T*_c_ and initial (black) and final pH (gray) levels from experiments with different initial acid concentrations. Data points are the mean and standard error from seven vesicles in a single experiment, and the line shows the average from the three independent experiments. The white scale bar on the images is equal to 100 μm.

A series of confocal images obtained from a typical experiment are shown in Figure 2b. There was a gradual increase in the fluorescence intensity in all vesicles following excitation at 458 nm, corresponding to an increase in pH as the reaction progressed. The coordinated transport of some vesicles, particularly at lower initial acid concentrations, was observed after the pH clock, possibly due to convection in the external solution (Figure 2c).^43^

The pH–time profile in seven individual vesicles is shown in Figure 2d. The rate of increase of pH in the vesicles was more gradual than that in aqueous-phase experiments. There was little evidence of a correlation between the vesicle diameter and the clock time (Figure S6) or between the spatial position of the vesicles and the clock time; the timing of the switch from pH 6 to 8 was similar in each vesicle. Overall, there was an increase in the clock time and a decrease in the initial pH with an increase in the initial acid concentration, as expected (SI 2.6 and Figure S7). The average clock time varied between repeats, but the standard deviation was small in each experiment, suggesting the possibility of a synchronized switch in pH in the vesicles mediated by the emission of ammonia (Figure 2e).

Propagating reaction–diffusion fronts were not observed in either the nano- or microvesicles under these conditions, probably as a result of the low concentration of vesicles. Hence, some insight can be gained from simulations with a simple ODE model of the vesicles and the external solution (SI 3.4). The reaction can be modeled by taking into account stochastic effects; however, in other work it was determined that population-level behavior was retained in the ODE models.^38,44^ The rate of change of the concentration of a species *A*_i_ in a vesicle was determined by the reaction and mass transfer rate as follows:^45−47^
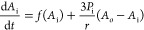
1where *f*(*A*_i_) contains the enzyme reaction and solution equilibria terms (SI 3.1–3.2) taken from earlier work,^10,11,48^*A*_0_ is the concentration of the species in the outer solution, *P*_i_ is the permeability coefficient of species i, and *r* is the radius of the vesicle. In the outer solution, the rate of change of the concentration of each species, *A*_0_, was given by the reaction rate (*g*(*A*_0_)) and the mass transfer rate (for identical vesicles) as follows:
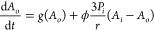
2where ϕ = *NV*_j_/*V*_0_ = the vesicle volume fraction, which takes into account dilution as a result of the volume change from vesicle to solution. The permeability coefficients for the neutral species were *P*_NH_3__ = 1 × 10^–4^ m s^–1^, *P*_CO_2__ = 1 × 10^–6^ m s^–1^, and *P*_Urea_ = 1 × 10^–8^ m s^–1^ are broadly in line with literature values (see SI 3.3).^47,49^ We assumed the permeability of the membrane to all ions (NH_4_^+^, CO_3_^2–^, HCO_3_^–^, H^+^, OH^–^, and pyranine) was negligible.

In experiments with nanovesicles of diameters ∼200 nm, the volume fraction of vesicles was estimated as ϕ = *NV*_i_/*V*_0_ ∼ 2 × 10^–3^ (see SI 1.6). A similar pH–time profile was obtained in the simulations with the enzyme concentration of [E] = 55 U mL^–1^, thus assuming an encapsulation efficiency of 25%.^50,51^ Initially, the pH increased rapidly in the vesicles, reaching a steady value around pH = 5.5 (Figure 3a, black curve). The pH switch in the vesicles at 15 min was accompanied by an increase in the pH of the outer solution (Figure 3a, red curve). The final pH in the vesicles was lower than that of the external solution as a result of the buffering effect of pyranine (Figure S8a) and the fact that the reaction was not at equilibrium at *T* = 90 min, as less than 2% of the urea was consumed (Figure S8b). The model was able to reproduce the experimental trends with changes in the initial acid concentration (Figure S9).

**Figure 3:**
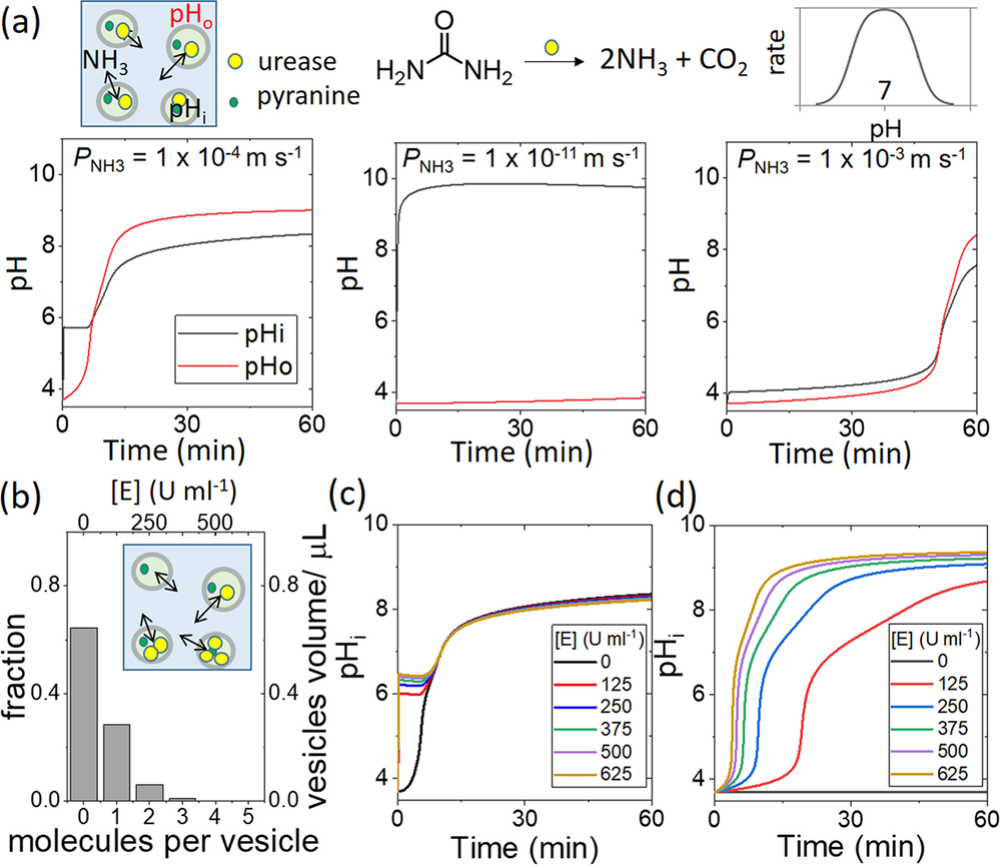
Simulations of the pH clock with a population of nanovesicles, *D* = 200 nm, with [E] = 55 U mL^–1^, [pyranine] = 5 mM, and [HCl] = 0.2 mM in a solution of [urea] = 50 mM and [HCl] = 0.2 mM with a vesicle volume fraction ϕ ∼ *NV*_i_/*V*_0_ = 2 × 10^–3^. (a) pH in time in identical vesicles (black curve, pH_*i*_) and the outer solution (red curve, pH_o_) and effect of the permeability coefficient of ammonia on the pH clock reaction. (b) Fraction of vesicles with *n* molecules of urease (Poisson distribution) and the equivalent enzyme concentration (U mL^–1^) for a given volume of vesicles (where ⟨E⟩ = 55 U mL^–1^, including empty vesicles, and a total vesicle volume of 1 μL). (c) Synchronized switch in pH for each volume fraction of vesicles given in panel b. (d) Range of pH clock times for each volume fraction of vesicles given in panel b with reduced ammonia permeability and enzyme turnover number (*P*_NH_3__ = 1 × 10^–11^ m s^–1^ and *k*_cat_′ = *k*_cat_/500).

The pH–time profile was mainly controlled by the transfer of ammonia to the outer solution (Figure S10). With a lower permeability coefficient of NH_3_, the pH increased rapidly in the vesicle to a high pH with no change in the outer solution, whereas for greater *P*_NH_3__ the pH in the vesicle and the external solution were the same (Figure 3a). The clock time increased to 90 min with *P*_NH_3__ = 1 × 10^–2^ m s^–1^, and the effect of encapsulation was eliminated. The same result could be obtained by having the enzyme dispersed in the external solution and ammonia diffusing into empty vesicles. This illustrates that compartmentalization played an important role in the pH–time profile in the vesicles, as the partial entrapment of ammonia raised the internal pH of the vesicles and enhanced the rate. Nevertheless, the switch was less sharp than that in aqueous-phase experiments because of the relatively fast loss of ammonia to the outer solution.

Simulations were performed with a distribution of the enzyme amount in the vesicles. On average, there was less than one enzyme molecule per vesicle (see SI 3.5.2). The probability of *n* molecules per vesicle was determined from a Poisson distribution (*P*(*X* = *n*), λ = 0.44), and the equivalent enzyme concentration in units per milliliter was determined from the total number of enzyme molecules in a given volume of the vesicles (Figure 3b). The average enzyme concentration for the heterogeneous population (including vesicles with no enzyme) was ⟨E⟩ = 55 U mL^–1^, and the clock time of *T*_c_ = 9.6 min was similar to that of the homogeneous population with [E] = 55 U mL^–1^ (*T*_c_ = 9.3 min). Vesicles with different enzyme loadings in the heterogeneous population have the same clock time despite the differences in their internal pH and enzyme concentration (Figure 3c). The potential impact of heterogeneity on the reaction can only be determined if we reduce both the membrane permeability to ammonia and the enzyme turnover number (*k*_cat_′ = *k*_cat_/500 to give the same average clock time of ∼9 min); then, a broad range of clock times can be observed (Figure 3d).

We also determined the influence of a distribution in enzyme loading and vesicle diameter on the pH clock reaction in the microvesicles. The vesicle volume fraction was ϕ = *NV*_j_*/V*_o_ = 0.018, and the number of enzyme molecules per vesicle was ∼10^5^ (SI 2.4). The encapsulation efficiency is generally assumed to be close to 100% using the droplet transfer method; however, significant differences in the macromolecular content have been reported.^34,35^ The simulations were undertaken using the experimental probability mass function along with a normal distribution for the enzyme concentration to obtain a bivariate histogram (SI 3.6) with a range of [E] = 60–100 U mL^–1^ and a diameter of 6–36 μm (Figure 4a). The membrane permeability to acetic acid (*P*_HA_) was also included in these simulations.

**Figure 4:**
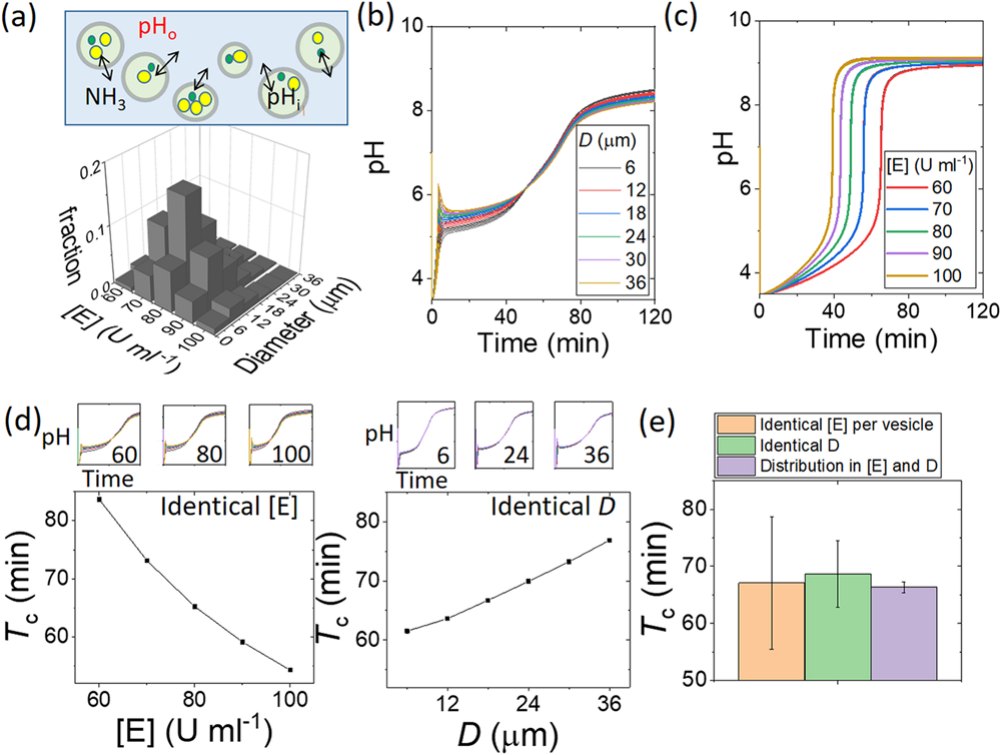
Effect of the urease concentration [E] and the vesicle diameter *D* on the pH clock in simulations of a population of microvesicles with [pyranine] = 50 μM in a solution of [urea] = 80 mM and [acetic acid] = 7.5 mM with a vesicle volume fraction ϕ = 0.018. (a) Bivariate distribution in the enzyme concentration with [E] = 80 U mL^–1^ and the vesicle diameter (*D*). (b) Synchronized switch in pH in vesicles with the distribution given in panel a. (c) Range of pH clock times in vesicles with both the distribution given in panel a and lower permeability and enzyme turnover number (*P*_NH_3__ = *P*_HA_ = 1 × 10^–11^ m s^–1^ and *k*_cat_′ = *k*_cat_/50). (d) Effect of [E] or *D* on the clock time *T*_c_ in simulations with a population of vesicles with either identical [E] per vesicle and the distribution in *D* given in panel a or identical *D* and the distribution in [E] given in panel a. (e) Comparison of the average and standard deviation (error bars) in the clock time for simulations in a population of vesicles with identical [E], identical *D*, or a distribution in both [E] and D.

The change in pH in the vesicles is shown in Figure 4b. The profiles are similar to those observed in experiments; there was a rapid increase in the internal pH to ∼5.5, then a transition to high pH at around 50 min that was accompanied by an increase in pH in the surrounding solution as a result of the fast transport of ammonia into the outer solution (Figure 4b(ii)). The initial pH was influenced by both the enzyme content and the diameter, with a smaller diameter and smaller enzyme concentration favoring low pH (Figure S11). Again, the synchronized switch in pH did not occur if the permeability coefficient of ammonia or acid and the enzyme turnover number were reduced; in that case, a range of clock times was obtained (Figure 4c).

The effect of the concentration of the enzyme, [E], on the pH clock with a high ammonia permeability was determined in simulations in which the enzyme concentration in each vesicle was identical but increased from 60 to 100 U ml^–1^ in separate runs. The clock time *T*_c_ decreased from 85 to 52 min as a result of the increased total amount of catalyst in the vesicles (Figure 4c). In simulations in which the diameter, *D*, of each vesicle was identical and increased from 6 to 26 μm in separate runs, the clock time increased from 60 to 80 min as a result of the reduced rate of transport of ammonia to the external solution (Figure 4c). In Figure 4d, the average clock times are shown for all the simulations with identical [E] or identical *D* (from Figure 4c), compared to the simulation with a distribution in both [E] and *D* (from Figure 4b). The standard deviation in *T*_c_ is small for the population with a bivariate distribution, as the pH switch is governed by communication between vesicles via the ammonia in the surrounding solution rather than the internal enzyme concentration or the diameter of each vesicle.

In conclusion, we have shown that the pH–time profile and the synchronization of the pH clock in heterogeneous vesicles was controlled by the relatively fast transport and increase in the amount of ammonia in the external solution. The behavior was observed in nano- and microvesicles with different phospholipids and acids, thus demonstrating the universal nature of the response. In natural systems, micro-organisms such as bacteria and yeast use extracellular signaling to overcome population diversity and change behavior.^52,53^ Ammonia is important in cell–cell communication and the multicellular structures that form in yeast and bacterial colonies. It has been implicated in complex functionalities such as metabolic oscillations and colony survival.^54^ Further control of the membrane permeability would provide a useful platform for the investigation of more complex collective behavior in populations of synthetic vesicles driven by acid or base changes.^55^

## Supporting Information Available

Preparation and analysis of the nanovesicles and microvesicles, including materials, urease encapsulation, calibration curve for pH, estimation of the number of vesicles, and kinetic data analysis with plots; modeling of the urease–urease reaction in vesicles, including the enzyme-catalyzed reaction, equilibria, transfer rates, equations and parameters; and model files (PDF)
