# Supplementary material for: Collective Behavior of Urease pH Clocks in Nano- and Microvesicles Controlled by Fast Ammonia Transport
Source: J Phys Chem Lett. 2022 Feb 21;13(8):1979–84. doi: 10.1021/acs.jpclett.2c00069 (PMC9007528; doi:10.1021/acs.jpclett.2c00069)
Supplement: Supplementary file 1 — jz2c00069_si_001.pdf [file jz2c00069_si_001.pdf]

Collective Behaviour of Urease pH Clocks in Nano- and Micro-vesicles Controlled by Fast Ammonia Transport

Ylenia Miele[1]#, Stephen J. Jones#[2], Federico Rossi[3] Paul A. Beales[2*] Annette F. Taylor[4*],*

1. Department of Chemistry and Biology, University of Salerno, Via Giovanni Paolo II, 132, 84084 Fisciano (SA), Italy.

2. School of Chemistry and Astbury Centre for Structural Molecular Biology, University of Leeds. Leeds LS2 9JT, UK.

3. Department of Earth, Environmental and Physical Sciences, University of Siena, Pian dei Mantellini 44, 53100 Siena, Italy.

4. Chemical and Biological Engineering, University of Sheffield, Sheffield S1 3JD, UK.

Supporting Information

Contents

1. Preparation and analysis of the synthetic nanovesicles....2

1.1 Materials....2

1.2 Urease-encapsulated nanovesicles....2

1.3 Phosphorus Assay....3

1.4 Calibration curve for pH....4

1.5 Estimation of number of vesicles....5

1.6 Nanovesicle Kinetic data analysis....5

2. Preparation and analysis of the synthetic microvesicles....8

2.1 Materials....8

2.2 Urease-encapsulated microvesicles 8
2.3 Confocal Imaging 9
2.4 Calibration curve for pH....9
2.5 Estimation of number of vesicles....9
2.6 Microvesicle Kinetic Data Analysis 10
3. Modelling of the urea-urease reaction in vesicles....11
3.1 Enzyme catalysed reaction 12
3.2 Equilibria....13
3.3 Transfer rates and permeability 14
3.4 Equations and parameters for populations of vesicles in solution 14
3.5 Nanovesicles....16
3.5.1 Homogeneous population 16
3.5.2 Heterogeneous population 17
3.6 Microvesicles....18
References....20
Appendix 1: Ode files for simulations in XPPaut 22

1. Preparation and analysis of the synthetic nanovesicles

1.1 Materials

The lipid, 1,2-diphytanoyl-*sn*-glycero-3-phosphocholine (DPhPC), and the fluorescent lipid marker (1,2-dioleoyl-*sn*-glycero-3-phosphoethanolamine-N-(lissamine rhodamine B sulfonyl) (ammonium salt)) (Rhod-DOPE), were purchased from Avanti Polar Lipids Inc. The ratiometric pH probe pyranine,

8-hydroxypyrene-1,3,6-trisulfonic acid trisodium salt (HPTS), urea and urease (Type III, 40 U mg^{-1}) from Canavalia ensiformis (Jack Bean) were purchased from Sigma-Aldrich and used without further purification.

1.2 Urease-encapsulated nanovesicles

The lipid film hydration and extrusion method was used to produce DPhPC + Rh-DOPE (0.5 mol%) liposomes of approximately 200 nm diameter. The phospholipid DPhPC was found to give more reproducible results in initial experiments with urease encapsulated in nanovesicles than palmitoyl-oleyl-phosphatidylcholine (POPC) used with the microvesicles.[1] DPhPC + Rh-DOPE thin films were formed by adding 300 μL of lipid in chloroform to a glass vial and drying overnight under vacuum. Hydrating solutions (1.0 mL) of different composition were used depending on the desired vesicle encapsulants for each experiment: for example, pyranine (0 or 20 mM), urease (Type III, 5.45 mg ml^{-1} = 220 U ml^{-1}) and HCl to adjust to the desired starting pH (e.g. 0.1 mM HCl for pH 4.0). Following vortex mixing, the samples were subjected to ten cycles of freeze-thawing (nine cycles at 45˚C, and the final cycle at 36˚C) to improve encapsulation efficiency.[2] It was verified that urease did not undergo significant degradation under these conditions. The samples were then extruded eleven times through a polycarbonate filter of pore size 200 nm to produce large unilamellar vesicles of approximately 200 nm, confirmed by Dynamic Light Scattering (DLS). Unencapsulated urease and pyranine were removed from the external medium by size-exclusion chromatography (Sephadex G50 medium) with a mobile phase corresponding to the pH of the encapsulated media (e.g. 0.1 mM HCl for pH 4.0) to produce a solution of nano-vesicles. The Rh-DOPE was used to visually track the vesicles in the column.

For Jack Bean urease Type III (Sigma-Aldrich) used in the experiments, the typical specific activity is 40 U mg^{-1} where 1 unit (U) = 1 μmol NH_3 min^{-1} at pH 7 and 25 ˚C. The molecular mass is M_r = 545 kDa (hexamer with Ni^{2+} included).[3] A concentration of 5.45 mg mL^{-1} (or 220 U mL^{-1}) corresponds to 10 μM urease assuming pure enzyme. However, Type III contains impurities including phosphates from

the purification process: purified urease has reported specific activities of >600 U mg^{-1} (Sigma-Aldrich Type C3) to 6000 U mg^{-1} (depending on the conditions of the assay e.g. temperature 20 - 38 ˚C, nature of buffer and pH).[4, 5] So the mass of urease in a Type III sample may be estimated from the ratio of the specific activity to pure, taken here as 40/600 x 100% = 6.7%, to give [E] = 0.7 μM. The encapsulation efficiency is <100% using the lipid hydration method, so this represents an upper limit in concentration.[6]

1.3 Phosphorus Assay

A phosphorus assay was used to determine the total phosphorus content (equivalent to the lipid concentration) of each vesicle solution produced. Six calibration samples were created by pipetting the following amounts of phosphorus standard solution (0.65 mM Phosphorus) into six borosilicate test tubes: 0 μmoles (0 μL), 0.0325 μmoles (50 μL), 0.065 μmoles (100 μL), 0.114 μmoles (175 μL), 0.163 μmoles (250 μL), and 0.228 μmoles (350 μL). Test samples were made by adding 50 μL of the liposome solution per test tube (in triplicate).

450 μL of 8.9 N H_2SO_4 was added to each test tube before heating in an aluminium block at 210 ˚C for 25 min. The test tubes were then removed from the block and allowed to cool for 5 min before addition of 150 μL of 30% w/w H_2O_2. The test tubes were placed back into the heating block for a further 30 min at 210˚C. The test tubes were then removed and allowed to cool for 5 min, before addition of 3.9 mL deionised water, 500 μL of 2.5% w/v ammonium molybdate (VI) tetrahydrate solution, and 500 μL of 10% w/v ascorbic acid solution. Each test tube was vortexed, and capped with parafilm, before being returned to the heating block for 7 minutes at 100˚C. The test tubes were removed from the heating block and allowed to cool to room temperature. The absorbance of each calibration standard and test sample was measured at 820 nm using a Cary 100 UV-vis spectrometer. A calibration curve was created from the standards and used to determine the phosphorous concentration of the liposome samples.

1.4 Calibration curve for pH

A calibration curve was produced for the indicator pyranine as a function of pH using a Cary 100 UV-Vis spectrophotometer. Pyranine exhibits a pH-dependent absorption and fluorescence spectrum and has been used for determination of pH in vesicles.[7, 8] The protonated form ($PyOH^{3-}$) has a λ_{max} at 405 nm and the deprotonated form (PyO^{4-}) at 457 nm (Figure S1). The ratio of absorbance, A, or fluorescence, F, of pH indicators at two wavelengths can be related to the concentration of free acid and the acid dissociation constant K_a.[9, 10] For pyranine:

$$PyOH^{3-} \rightarrow PyO^{4-} + H^+ \qquad K_a = [PyO^{4-}][H^+]/[PyOH^{3-}] \qquad \text{(SE1)}$$

$$R = R_{min} + \frac{R_{max} - R_{min}}{1 + 10^{(\mathrm{pKa}' - \mathrm{pH})}} \qquad \text{(SE2)}$$

$$\mathrm{pH} = \mathrm{pKa}' - \log\left(\frac{R - R_{max}}{R_{min - R}}\right) \qquad \text{(SE3)}$$

where R is the ratio of absorbances: A_{450}/A_{405}, R_{min} and R_{max} are the asymptotic limits of the curve at low and high pH (R_{min} is the ratio of absorbance of the protonated species ($PyOH^{3-}$) at the two wavelengths, R_{max} is the ratio of absorbance of the deprotonated form (PyO^{4-}) and K_a' is the apparent dissociation constant of pyranine which takes into account an additional absorbance factor. Ratiometric measurements were used to ensure the data is independent of the concentration of fluorophore and we used the ratio of absorbances here rather than ratio of fluorescences as the calibration curve was found to be more sensitive to changes in pH at higher pH, giving a more accurate determination of the final pH of the pH clock reaction. However the absorbance measurements resulted in greater error at low pH.

A phosphate-citrate buffer, covering a pH range of 4.0 – 8.0, and a glycine-NaOH buffer, covering a pH range of 8.6 – 10.6, were used to produce the calibration curve. The absorbance of each buffered solution containing pyranine (50 μM) and deionised water-encapsulating liposomes of approximate diameter 200 nm at a phosphorus content of 250 μM was measured at room temperature. A fit to the

data was obtained in OriginPro using the equation: $y = a + (b - a)/(1 + 10^{d*(c - x)})$ from SE2 with the addition of the parameter d to give a better fit to the data at low pH as the presence of liposomes resulted in an x-axis offset (Figure S1(b)). The (apparent) pH was determined from SE3: $pH = c - (1/d)\log((y - b)/(a - y))$ and, using the formula for the propagation of errors, the error in the pH, s_{pH}, was related to the error in R (s_y): $s_{pH} = (s_y^2((y - b)/(y - a)^2 - 1/(y - a))^2(y - a)^2)/(d^2(y - b)^2))^{1/2}/\ln(10)$.

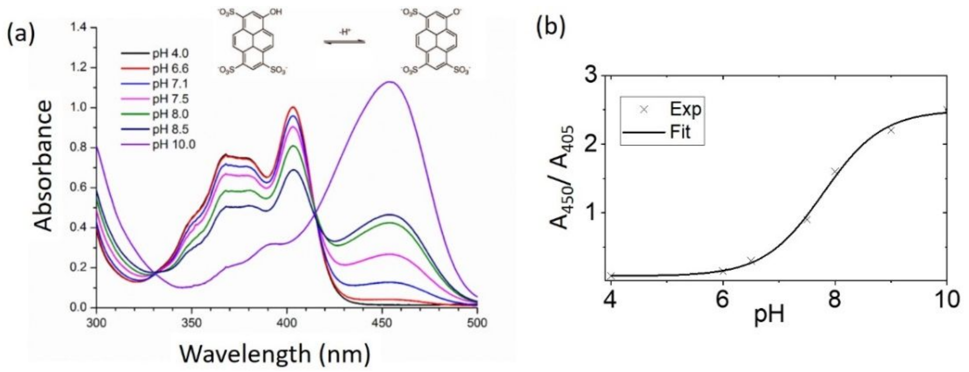

Figure S1. Calibration curve for pyranine as a function of pH. (a) Absorption spectrum of 50 μM pyranine in phosphate-citrate or glycine-NaOH buffer solution and corresponding pH. (b) Ratio of absorbance, 450 nm/405 nm, vs pH in buffer solution with pyranine and 200 nm vesicles (points) and fitted equation (line): $y = a + (b - a)/(1 + 10^{d*(c-x)})$ where a = 0.08 ± 0.06, b = 2.49 ± 0.08, c = 7.79 ± 0.07 and d = 0.82 ± 0.12.

1.5 Estimation of number of vesicles

The total number of lipids per vesicle is given by the surface area of the inner and outer monolayers of the unilamellar vesicle divided by the head group of a single lipid molecule:

$$N_{tot} = \frac{\left[4\pi\left(\frac{D}{2}\right)^2 + 4\pi\left[\frac{D}{2} - h\right]^2\right]}{a} \qquad \text{(SE4)}$$

where D is the diameter of the vesicle and h is the bilayer thickness. For DPhPC (M_r = 846 mol g^{-1}), h = 4 nm and a = 0.78 nm^2.[11] With 200 nm vesicles, this gives $N_{tot} = 3.1 \times 10^5$ vesicle^{-1}. Samples were diluted to 250 μM phosphorous concentration in the total sample volume of V_o = 500 μL. The total

molecules of phospholipid = cV_oN_a = 7.5 x 10^{16}. So the total number of vesicles is of the order of N = 7.5 × 10^{16}/3.1 x 10^5 = 2.4 × 10^{11} and number density, $n = N/V_o$ ~ 4.8 x 10^8 vesicles μL^{-1} = 4.8 x 10^{17} m^{-3}. The estimated (outer) volume of the vesicle was ~ V_j = 4.2 x 10^{-12} μL and the vesicle volume fraction was estimated as $\phi = NV_i/V_o$ = 2 x 10^{-3}, with total volume of vesicles = 1.0 μL.

1.6 Nanovesicle Kinetic data analysis

Vesicle solutions were diluted to 500 μM lipid concentration. A volume of 250 μL was placed in a 550 μL micro-cuvette (2 x 3.5 mm window, path length 10 mm) and 250 μL urea (100 mM) in hydrochloric acid solution was added to initiate the reaction. Measurements were typically collected using a Cary 100 UV-Vis spectrophotometer every minute for the first 60 minutes then every five minutes for each kinetic run. All experiments were performed at room temperature (20 ± ˚C).

The data was analysed using OriginPro. Representative curves for individual runs with different acid concentration are shown in Figure S2(a). In order to determine a clock reaction time, a Hill fit was produced of the absorption ratio, $R = A_{450}/A_{405}$, in time, t, for each kinetic run: $R = a + (b - a)t^n / (k^n + t^n)$ where k = width at half max, a is the minimum absorption ratio, b = the maximum ratio absorption. The pH in time (Figure S2(b)) was determined using the experimental value of R and equation SE3. There was a large variation in the initial pH (< 5 mins), probably as a result of mixing of the urea and vesicle solution, and the final pH was much lower than in the aqueous phase experiments, where the clock time is usually taken as time to the maximum rate or pH = 7. Hence, the clock time T_c was defined here as the time to reach R = 0.3748 where pH = 6.75.

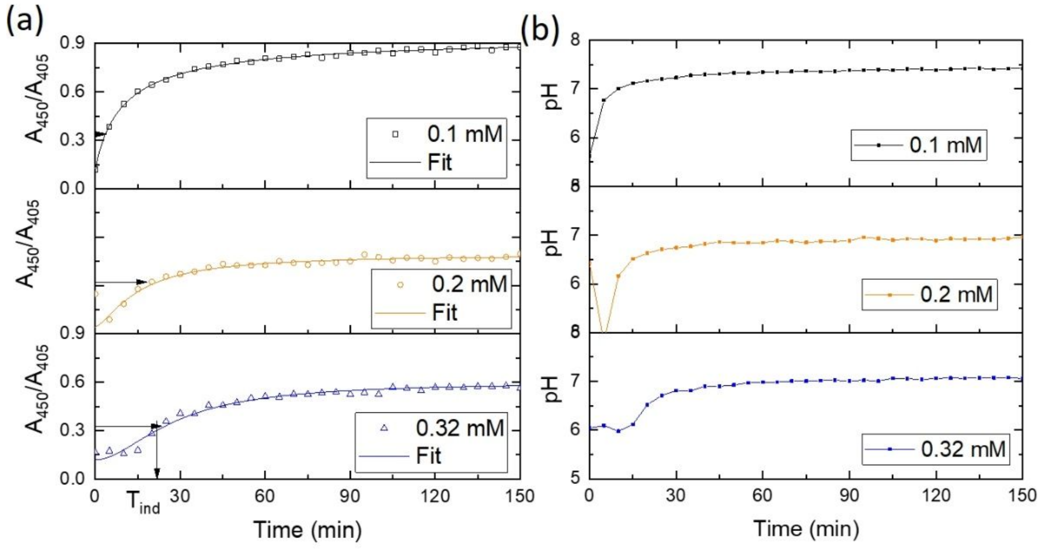

Figure S2. Urease reaction in vesicle solution prepared with hydrating solution: urease (220 U mL^{-1}), HCl (0.1 – 0.32 mM) and pyranine (20 mM) and placed in a microcuvette to which a solution of urea (100 mM) and HCl (0.2 – 0.64 mM) was added to initiate the reaction. (a) Ratio of absorbance in time for different acid concentrations and Hill fit with equation $y = a + (b - a)x^n / (k^n + x^n)$ used to determine the time to reach $R = 0.3748$ or pH = 6.75. (b) Apparent pH in time determined from curves shown in (a) and pH calibration curve.

In order to determine whether any unencapsulated enzyme from, for example, burst vesicles, in the solution could influence the reaction, a control experiment was performed in which surfactant Triton-X was added to lyse the vesicles. Upon addition of Triton-X to the solution, with vesicles and urea no change in absorbance was observed (Figure S3), demonstrating that the total amount of enzyme contained in the vesicles was insufficient to catalyse the reaction once diluted in outer solution.

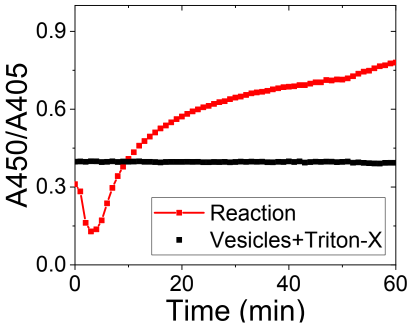

Figure S3. Ratio of absorbances for urease reaction in vesicles and an equivalent experiment with Triton-X added to rupture the vesicles. Vesicles were prepared with hydrating solution: urease (220 U ml^{-1}), HCl (0.2 mM) and pyranine (20 mM) and placed in a microcuvette to which a solution of urea (100 mM) and HCl (0.4 mM) was added to initiate the reaction.

Data was collected from three independent experiments for each value of the initial concentrations. The mean ratio of absorbance, R, was determined in time and the corresponding standard errors were calculated for Figure 1(b) and the mean value of R was used to determine the pH and error in pH for Figure 1(c). The error in the pH was determined using propagation of errors, using the standard error of the ratio of absorbance. The mean and standard error of T_c were determined from the Hill fit of the three independent experiments. The initial and final value of the pH at 5 mins and 90 mins were determined from the value of R from the fitted relationship and equation 3. The initial pH data was taken at 5 mins to avoid the influence of initial mixing of the urea solution and the vesicles but was also unreliable as a result of the large error at low pH (Figure S4).

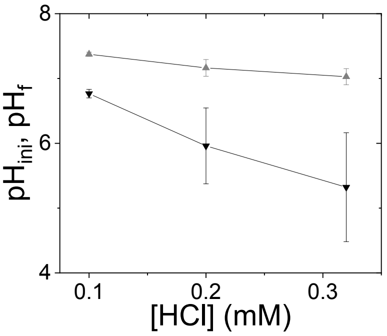

Figure S4. Average initial pH_{ini} (5 min) and final pH_f (90 min) for three kinetic runs with the nanovesicles at different initial acid concentration. Vesicles were prepared with urease (220 U ml^{-1}), HCl (0.2 mM) and pyranine (20 mM) and a solution of urea (100 mM) and HCl (0.2 – 0.64 mM) was added to initiate the reaction. Error bars show the standard error from 3 independent experiments.

2. Preparation and analysis of the synthetic microvesicles

2.1 Materials

Urea (Sigma-Aldrich), urease (Type III Jack bean U1500-20KU, Sigma-Aldrich, of specific activity 40.3 U mg^{-1}), pyranine (Sigma-Aldrich), the phospholipid 1-palmitoyl-2-oleoyl-*sn*-glycero-3-phosphocholine (POPC, Lipoid), mineral oil (M5904, Sigma-Aldrich), acetic acid (Sigma-Aldrich), glucose and sucrose (Carlo Erba) were of analytical grade and used without further purification.

2.2 Urease-encapsulated microvesicles

The microvesicles were prepared using a water-in-oil (w/o) emulsion droplet transfer method, which typically produces a population of giant unilamellar vesicles (GUV) of sizes 1 – 100 μm.[12, 13] Ultrapure water was used for preparation of solutions. Aqueous enzyme and sugar solutions were freshly prepared and used the same day. Lipid-in-oil solutions were kept refrigerated for up to one week. The lipid-in-oil solution was prepared by diluting a suspension of POPC (3 mM) in mineral oil (MO) to produce a solution of POPC (0.5 mM) in MO.

An oil-water interface was produced by adding 300 μL of the lipid-oil solution to 500 μL of an aqueous solution, the so-called outer solution (O-solution), which includes glucose (0.2 M) and acetic acid (1 – 7.5 mM). Next an aqueous inner solution, (I-solution), was prepared by mixing sucrose (0.2 M) with pyranine (50 μM) and urease (80 U mL^{-1}). A lipid-stabilized water-in-oil emulsion was prepared by emulsifying 20 μL of the I-solution in 600 μl of lipid-oil solution ([POPC] = 0.5 mM) by pipetting the solution 20 times with a P1000 micropipette set at 500 μL. The water-in-oil emulsion was poured above the oil-water interface. The I- and O-solutions are isotonic, but their densities are different, for sucrose 1.24 g ml^{-1}, for glucose 1.12 g ml^{-1}. We facilitated the droplet transfer by centrifuging the system for 10 min at 6000 rpm (200 g). After centrifuging, the supernatant was poured out and the pellet of vesicles

was washed with repeated addition of O-solution to remove the excess solutes and ensure no enzyme was present in the outer solution.

Vesicles were prepared with a solution of urease Type III (40.3 U mg^{-1}) at 80 U mL^{-1} or 2.0 mg mL^{-1}. With M_r = 545 kDa = 545 x 10^3 g mol^{-1}, this corresponds to 3.7 μM assuming pure enzyme, however, Type III contains impurities: pure urease has reported specific activities of >600 – 6000 U mg^{-1} (depending on the conditions of the assay, temperature, nature of buffer and pH).[4, 14] So, the mass of urease in a sample may be estimated as 40/600 x 100% = 6.7%, to give [E] = 0.25 μM.

2.3 Confocal Imaging

Images of the vesicles were obtained with a Leica TCS SP8 confocal microscope with a 20X objective (HC PL APO CS2 20x/0.75 DRY). The images were 1024 x 1024 pixels with a field of view of 406.47 x 406 μm (2.5 pixels/μm) and the optical thickness was 56.6 μm. For the ratiometric calculations, two excitation lasers were used: the 405 diode and 458 nm Argon. The emission wavelength range was 485 – 555 nm. Images were obtained every 42.6 s in each channel. The scan speed was 400 Hz and the line averaging was 8.

2.4 Calibration curve for pH

Lipid vesicles containing different pH buffer solutions and pyranine (50 μM) were prepared to obtain a pH calibration curve. The range of pH between 4.6 and 8.2 was covered with a citrate-phosphate buffer, pH 9.26 and 10.06 with an ammonia/ammonium buffer. The total concentration of the ions inside the vesicles was [HPO_4^{2-}] + [citric acid] = 0.0015 M and [NH^{4+}] + [NH_3] = 0.002 M. The images were analysed using MATLAB to obtain the average intensity of each vesicle and ratio of fluorescence intensities shown in Figure S5. The calibration curve is given by (SE2): $R = a + (b - a)/ (1+10^{(c - pH)})$ where R is the ratio of fluorescence, and the pH is given by (SE3): $pH = c + \log(R - a)/(b - R)$. There

are some structural differences in the microvesicles (eg surface attached lipids, formation of multisomes) that likely contribute to the error in the ratio of fluorescence at a given pH.

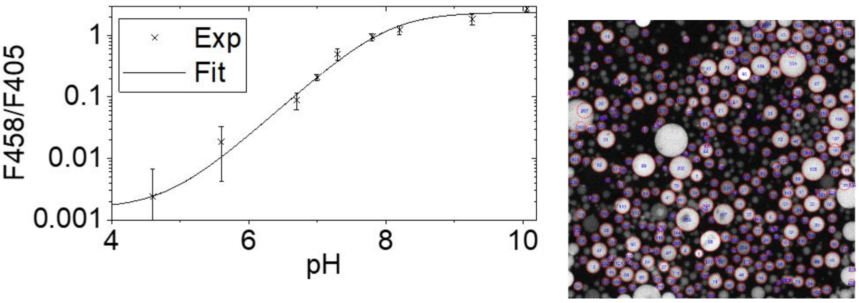

Figure S5. Calibration curve for pyranine in microvesicles. Experimental measurements (points) of ratio of fluorescence as a function of pH for vesicles prepared using buffer solutions and fitted equation (line): $y = a + (b - a)/(1+10^{(c - pH)})$ with the fitted parameters: $a = 0.00179 \pm 0.00352$, $b = 2.31 \pm 0.20$, $c = 8.01 \pm 0.06$. Error bars correspond to standard deviation from two separate samples and total number of vesicles $n > 400$. Right panel shows the vesicles in a field of view of 406 x 406 μm^2.

2.5 Estimation of number of vesicles

A tile scan was performed of the entire reactor chamber and the total number of vesicles was of the order of $N = 3 \times 10^4$ which is a lower estimate of the total amount of vesicles It was noted that there was some spatial variation in density. In a typical kinetic run, images were obtained every 40 s, hence it was impractical to monitor the whole reaction chamber, and 0.4 mm x 0.4 mm images were obtained. Thus, to establish a representative number density in a typical kinetic run, the number and diameter of vesicles from images of nine separate kinetic runs was determined using MATLAB, with a radius bigger than 5 pixels (2 μm). The data was used to produce a probability mass function for the vesicle diameter (Figure 2a). The total volume of 1024 vesicles from nine runs was 0.00272 μL and the total volume of solution in the nine runs was $V_o = 0.151$ μL. The average number of vesicles in an image was $N = 113 \pm 43$. This gives number density $N/V_o = 7.1 \times 10^{12}$ vesicles m^{-3} and volume fraction $\phi = NV_j/V_o = 0.018$.

2.6 Microvesicle Kinetic Data Analysis

The reaction chamber was constructed using a transparent silicone sheet (MXBAOHENG, thickness 0.2 mm) with a circular hole of diameter 10 mm and placed on a microscope slide. For the reaction, 20 μL of vesicles in O-solution ([HA] = 1 – 7.5 mM) was added to 10 μL of urea/acid solution ([urea] = 0.08 M, [HA] = 1 - 7.5 mM), mixed and then 30 μL was added to the microscope slide and sealed with the cover slip, ensuring no air gap. The mixing time was of the order of three minutes. Reactions were performed at room temperature, 20 ± 2 ˚C. Images were processed using MATLAB to find the size and position of each vesicle and the average intensity per vesicle was determined from the sum of the pixel intensities divided by the area of the vesicle. The intensity in time was recorded from both the 458 nm and 405 nm excitation and the ratio of intensities calculated.

The switch in pH in individual vesicles is shown in the space-time plot in Figure S6(a), which was constructed from data along the line shown in the image of the vesicles. The increase in fluorescence was clearly visible at $T = 60$ min in all the vesicles. Three of the vesicles remained stationary after the pH clock, while one can be observed to undergo motion i.e. the diagonal line. The vesicle diameter is plotted along with the clock time in each vesicle and initial and final pH in Figure S7(b). There was little evidence of a correlation between diameter and initial or final pH.

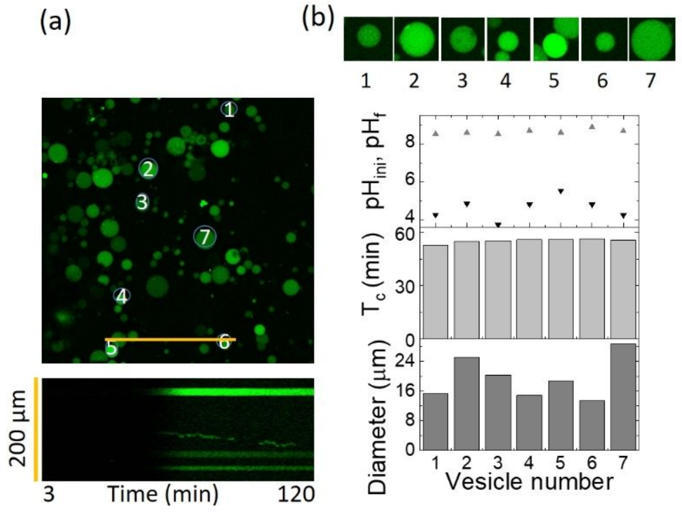

Figure S6 (a) Confocal image (460 μm x 460 μm) of the seven vesicles used in data analysis for 7.5 mM acid; orange line corresponds to the space-time plot below the image (b) diameter of vesicles and corresponding clock time, T_c, and initial and final pH in each vesicle from experiment shown in (a).

Data is presented from three independent experiments for each set of initial concentrations. For each experiment, the ratio of fluorescence, *R*, in time was determined for seven vesicles. Representative plots of *R* and the corresponding pH with different initial acid concentration are shown in Figure S7. Hill fits with $R = a + (b - a)t^n / (k^n + t^n)$ where k = width at half max, a is the minimum absorption ratio, b = the maximum ratio absorption were used to determine the clock time for each individual vesicle. The clock time T_c was defined as the time to reach $R = 0.206$ where pH = 7.0, and the value b was used to determine the pH_f using the fitted relationship (Figure S6). The mean T_c, initial pH (T = 5 mins) and final pH and standard deviations for the seven vesicles were calculated.

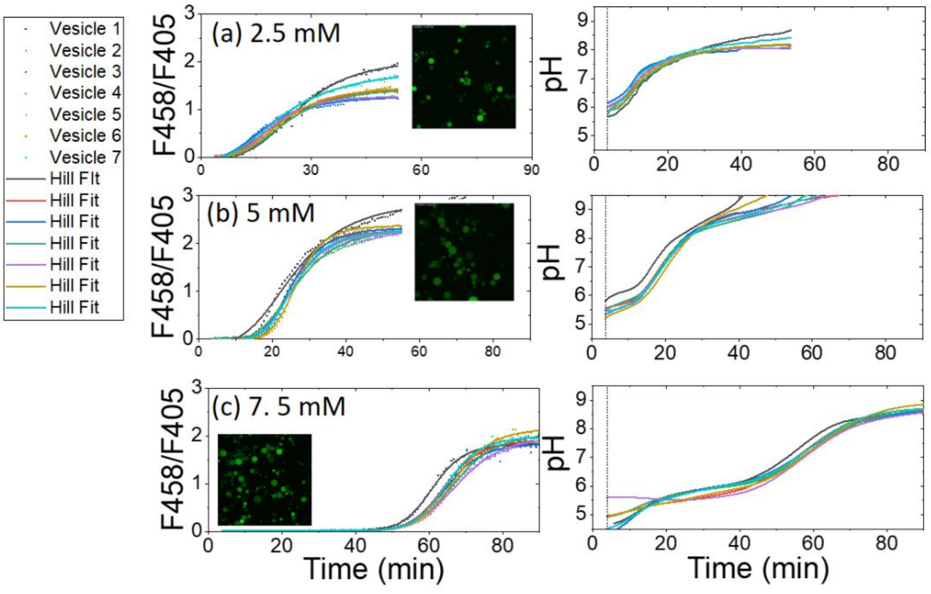

Figure S7. Representative kinetic runs in the microvesicles with different initial acid concentrations. The ratio of fluorescence at 458 nm to 405 nm and corresponding pH were determined for 7 vesicles in the confocal images (inset) of the reaction.

3. Modelling of the urea-urease reaction in vesicles

The system was modelled as a set of coupled ordinary differential equations (ODEs) describing the rate of change of species in the vesicles and the external solution, hence assuming the solutions are well-mixed on the length scales considered. Diffusion is fast compared to reaction in the vesicles: the diffusion timescale of ammonia in a 20 μm vesicle, with a diffusion coefficient of 2×10^{-3} mm^2 s^{-1} at 298 K,[15] is $t = L^2/D = 0.2$ s. On greater length scales, the urease reaction can support propagating pH fronts in thin layers of solution with speeds of the order of 0.5 mm min^{-1}, providing the total enzyme concentration is above a threshold amount (> 1 U ml^{-1} at pH 4).[16] The rate of change of pH in experiments was generally much slower than in our earlier work and there was no evidence of pH fronts in the experiments presented here, probably because the vesicle volume fraction was too low.

The main processes in the model are (3.1) the enzyme-catalysed reaction in the vesicles, (3.2) the equilibria that govern the pH in the vesicles and the surrounding solution and (3.3) the mass transfer of molecules between the vesicles and external solution. The ODE model used here takes into account all 12 chemical species in reactions R1 – R7 (not including water) to determine pH more accurately.

3.1 Enzyme catalysed reaction

The enzyme catalysed hydrolysis of urea produces ammonia and carbon dioxide:

(R1) $$CO(NH_2)_2 + H_2O \xrightarrow{urease} 2NH_3 + CO_2$$

where the rate of the enzyme catalysed reaction is given by a modified Michaelis-Menten equation (for simplicity acid is included as H^+ rather than H_3O^+):[3, 17]

$$v = \frac{k_{cat}[\mathrm{E}]_\mathrm{T}[\mathrm{urea}]}{(K_M + [\mathrm{urea}])\left(1 + \frac{[\mathrm{urea}]}{K_s}\right)\left(1 + \frac{[\mathrm{NH_4^+}]}{K_p}\right)\left(1 + \frac{K_{es2}}{[\mathrm{H^+}]} + \frac{[\mathrm{H^+}]}{K_{es1}}\right)} \quad \text{(SE5)}$$

and k_{cat} is the turnover number (s^{-1}), $[E]_T$ is the concentration of enzyme in the vesicle (mol dm^{-3}, or M), K_M is the Michaelis constant, K_{es2} and K_{es1} are protonation equilibria of the substrate-enzyme complex. Substrate and product inhibition terms were included: K_s = equilibrium constant for uncompetitive substrate inhibition and K_p = equilibrium constant for non-competitive product inhibition.

The urease enzyme Type III (Sigma-Aldrich) used in experiments is not pure and has specific activity of 40 U mg^{-1} (1 unit = 1 μmol NH_3 min^{-1} at pH 7 and 25 °C) compared to 600 – 6000 U mg^{-1} of pure urease. In order to facilitate comparison of the simulations with experiments, we used U mL^{-1} for the enzyme concentration:

$$[E]\ (U\ ml^{-1}) = m \times s \qquad \text{(SE6)}$$

where s is the specific activity and m is the mass concentration of Type III, in mg mL^{-1}. The concentration of urease, $[E]_T$, in M, can be estimated from the activity of Type III relative to the activity of pure urease, p, and the molecular mass of Jack Bean urease, M_r = 545000 g mol^{-1}:

$$[E]_T(M) = m\frac{s}{p}\frac{1}{M_r} \qquad \text{(SE7)}$$

Then the maximum enzyme rate, v_{max}, in M s^{-1}, is given by:

$$v_{max} = k_{cat}[E]_T = k_{cat}m\frac{s}{p}\frac{1}{M_r} \qquad \text{(SE8)}$$

So to account for the enzyme concentration in U mL^{-1};

$$v_{max} = k_{cat}\frac{[E]}{s}\frac{s}{p}\frac{1}{M_r} = k_{cat}[E]\frac{1}{pM_r} \qquad \text{(SE9)}$$

We set $k_1 = k_{cat}/pM_r$ so that $k_{cat}[E]_T = k_1[E]$ where [E] is in U ml^{-1} and (as mg g^{-1} is equivalent to ml L^{-1}) k_1 has units of M U^{-1} ml s^{-1}. Turnover numbers for Jack Bean urease are reported as $k_{cat} = (1 – 90) \times 10^3$ s^{-1} (pH 5.5 - 8 and 15 - 38 °C).[4, 18, 19] We took $k_1 = 3.6 \times 10^{-6}$ M U^{-1} ml s^{-1} in line with our earlier

work[20] under nonbuffered conditions at 20 ˚C and with $p = 600$ U mg^{-1} (Sigma-Aldrich Type C3), $k_{cat} = 1.2 \times 10^3$ s^{-1}.

Experiments with urease encapsulated in egg lecithin liposomes suggested that there was no change in the maximal enzyme rate as a result of the encapsulation under buffered conditions,[6] and variation of the enzyme constants did not have a significant effect on the overall trends reported here. The values of all the enzyme constants are given in Table 1.

3.2 Equilibria

The pH inside and outside the vesicles is determined by the following reversible reactions:

(R2) $NH_4^+ \rightleftharpoons NH_3 + H^+$ pK$_a$ = 9.25, rate = $k_2[NH_4^+] - k_{2r}[NH_3][H^+]$

(R3) $CO_2 + H_2O \rightleftharpoons H^+ + HCO_3^-$ pK$_a$ = 6.35, rate = $k_3[CO_2] - k_{3r}[HCO_3^-][H^+]$

(R4) $HCO_3^- \rightleftharpoons H^+ + CO_3^{2-}$ pK$_a$ = 10.25, rate = $k_4[HCO_3^-] - k_{4r}[CO_3^{2-}][H^+]$

(R5) $H_2O \rightleftharpoons H^+ + OH^-$ pK$_a$ = 14, rate = $k_5 - k_{5r}[OH^-][H^+]$

The protonation of the indicator, pyranine (HPTS), in the vesicles was accounted for:

(R6) $PyOH^{3-} \rightleftharpoons PyO^{4-} + H^+$ pK$_a$ = 7.39 rate = $k_6[PyOH^{3-}] - k_{6r}[PyO^-][H^+]$

The desorption of gaseous CO_2 or NH_3 from the surrounding solution are not included here nor H_2CO_3 as this species rapidly forms CO_2. In the presence of acetic acid in the experiments with microvesicles, the following reaction was taken into account:

(R7) $CH_3COOH \rightleftharpoons CH_3COO^- + H^+$ pK$_a$ = 4.75 rate = $k_7[HA] - k_{7r}[A^-][H^+]$

The acid equilibria rate constants are well established.[21] The pyranine pK$_a$ varies depending on the ionic strength.[22]

3.3 Transfer rates and permeability

The transfer of species across the membrane boundary was assumed to follow a simple solubility-diffusion mechanism:

$$J_i = -P_i \Delta C \qquad \text{(SE10)}$$

Where J_i is the flux of the solute species i, P_i is the permeability coefficient (m s^{-1}) that depends on the nature of the membrane and the chemical species, and ΔC is the concentration drop across a membrane of thickness L. The rate of change of concentration in a spherical vesicle of surface area A and volume V was thus:[23]

$$\frac{dC}{dt} = \frac{A}{V} P_i \Delta C = \frac{3P_i}{r} \Delta C \qquad \text{(SE11)}$$

The permeability coefficients are related to the diffusion coefficient and partition coefficient of the species in the organic phase. The transfer of neutral species: urea, ammonia, carbon dioxide and acetic acid were included here. The values of the permeability coefficients given in the literature vary depending on the method of determination and may be hindered by unstirred layer effects.[24] We set $P_{NH3} = 1 \times 10^{-4}$ m s^{-1}, and $P_{CO2} = 1 \times 10^{-6}$ m s^{-1} and $P_{Urea} = 1 \times 10^{-8}$ m s^{-1} , broadly in line with literature values.[25, 26] The permeability of acetic acid was taken as $P_{HA} = 1 \times 10^{-7}$ m s^{-1}.[27] Permeability coefficients of neutral species are unlikely to vary greatly between the two lipids of similar carbon chain length (POPC and DPhPC) used here.

The transfer of ionic species and large species was assumed to be negligible on the timescale of the experiment (~ 1 hour). Proton permeability is controversial and has wide ranging values reported from 10^{-11} – 10^{-3} m/s.[28] Proton transport may involve a fast initial component that is quickly balanced by formation of an electrical gradient. Here, the permeability of protons, (P_{H+}) was taken as zero.

We did not take into account osmosis in these simulations. In general, we did not observe significant change in volume of the vesicles during the course of an experiment with microvesicles, however water flow may contribute to some of the behaviour and will be explored in future work.

3.4 Equations and parameters for populations of vesicles in solution

In the vesicles, the rate of change of concentration of a species A_i is determined by the reaction rate and net transfer rate (where applicable):

$$\frac{dAi}{dt} = f(A_i) + \frac{3P_i}{r}(A_o - A_i) \qquad \text{(SE8)}$$

Where $f(A)$ contains all the relevant reaction terms (included in the code in Appendix), P is the permeability coefficient, r is the radius of the vesicle and A_o is the concentration in the outer solution. The rate of change of concentration of each species in the surrounding solution is given by the reaction terms and the sum of moles of species transferred to the surrounding solution from each vesicle per unit time, divided by the volume, V_o, of outer solution. For $j \ldots N$ vesicles:

$$\frac{dAo}{dt} = g(A_o) + \frac{1}{V_o}\sum_j^N V_j \frac{3P_i}{r}(A_o - A_i) \qquad \text{(SE9)}$$

where $g(A_o)$ contains the relevant reaction terms in the surrounding solution and V_j is the volume of the jth vesicle. For identical vesicles this reduces to:

$$\frac{dAo}{dt} = g(A_o) + \phi \frac{3P_i}{r}(A_i - A_o) \qquad \text{(SE10)}$$

where $\phi = N\, V_j/V_o$ – the ratio of the total volume of vesicles to the total volume of solution, i.e. the vesicle volume fraction or vesicle concentration (v/v). The values of all the rate constants and permeability coefficients taken in this work are shown in Table 1. The rate equations were solved using XPPAUT with integration method STIFF.[29] The ode file for the models used in the simulations presented here are included in the Appendix.

Table 1. Rate constants (20°C) and permeability coefficients for urease reaction in vesicles.

Equilibria rate constants	k_2 s^{-1}	k_{-2} M^{-1} s^{-1}	k_3 s^{-1}	k_{-3} M^{-1} s^{-1}	k_4 s^{-1}	k_{-4} M^{-1} s^{-1}
	24	4.3×10^{10}	0.037	7.9×10^4	2.8	5×10^{10}
	k_5 M s^{-1}	k_{-5} M^{-1} s^{-1}	k_6 s^{-1}	k_{-6} M^{-1} s^{-1}	k_7 s^{-1}	k_{7r} M^{-1} s^{-1}
	1×10^{-3}	1×10^{11}	1	2.5×10^7	7.8×10^5	4.5×10^{10}
Enzyme constants	k_1 M U^{-1} ml s^{-1}	K_M M	K_{es1}	K_{es2}	K_s	K_p
	3.6×10^{-6}	3×10^{-3}	5×10^{-6}	2×10^{-9}	3	0.02
Permeability coefficients (m s^{-1})	P_U	P_{NH3}	P_{CO2}		P_{CHOOH}	
	1×10^{-8}	1×10^{-4}	1×10^{-6}		1×10^{-7}	

3.5 Nanovesicles

3.5.1 Homogeneous population

The XPPAUT file is given in Appendix, model 1. The vesicle diameter was 200 nm, and the vesicle volume fraction was $\phi = NV_i/V_o = 2 \times 10^{-3}$. The initial concentrations unless otherwise stated were: [E] = 55 U ml^{-1}; [$PyOH^{3-}$] = 5 mM, and [H^+] = 0.1 – 0.32 mM. Outer Solution: [Urea] = 50 mM; [H^+] = 0.1 – 0.32 mM. All other concentrations were set equal to zero.

The buffering effect of pyranine on the urease pH clock reaction in a vesicle, with no mass transfer, is seen in Figure S8(a). The concentration of urea is reduced by less than 2% as a result of the pH switch in the vesicles, thus the reaction is not at equilibrium at T = 90 min (Figure S8(b)).

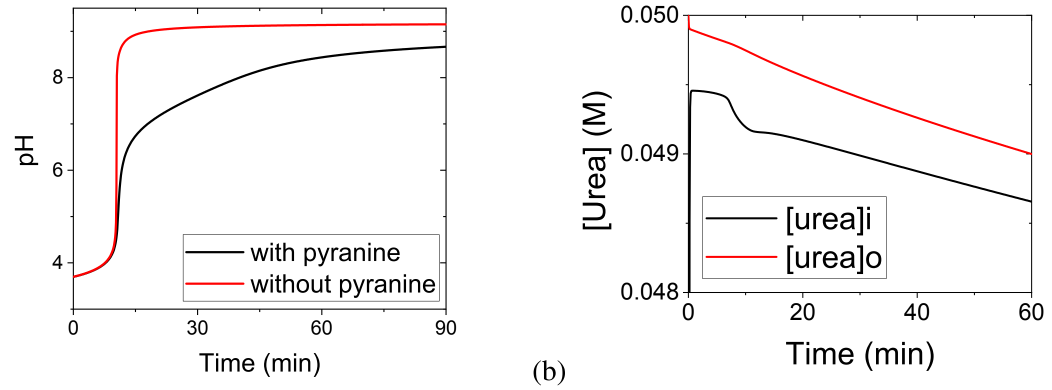

(a) (b)

Figure S8. (a) Effect of pyranine on urease pH clock in a vesicle with no mass transfer ($P = 0$) with [E] = 1 U ml^{-1}, [$PyOH^{3-}$] = 5 mM, [Urea] = 50 mM, [HCl] = 0.2 mM. (b) Consumption of urea during the clock reaction in a vesicle with mass transfer [E] = 55 U ml^{-1}, [HCl] = 0.2 mM. The internal concentration of urea shows an acceleration in the rate of loss as a result of the pH feedback during the pH clock.

The pH time profile is shown in Figure S9(a) for the reaction in vesicles at three different initial acid concentrations and the corresponding clock time, T_c, (to pH = 6.75) and initial (5 min) and final pH (90 min) as a function of acid concentration are shown in Figure S9(b).

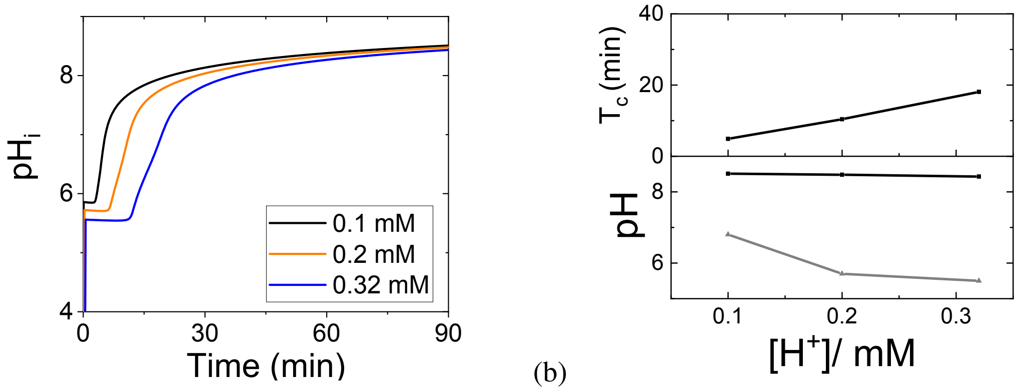

(a) (b)

Figure S9. (a) pH-time profiles for urease reaction in the vesicles at different initial acid concentrations (b) Clock time, T_c, and initial (lower curve, grey) and final (upper curve) pH as a function of initial acid concentration.

The ammonia produced in the vesicle was protonated forming NH_4^+ which was trapped in the vesicle lumen thereby increasing the internal pH and the rate of the enzyme reaction. The ammonium acted as a supply of NH_3 which was transferred out as fast as it is produced, resulting in a steady value in the vesicle, and a slowly increasing concentration in the outer solution. (Figure S10).

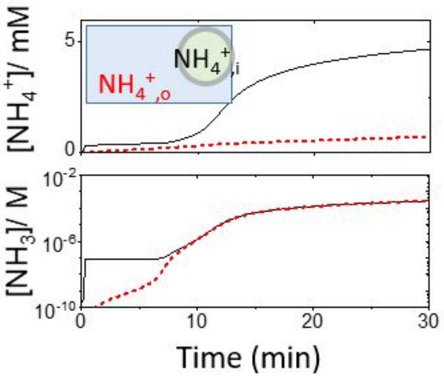

Figure S10. Concentration of ammonium and ammonia in the vesicles (black, solid) and the outer solution (red, dotted) in time. Simulations with urease [E] = 55 U mL^{-1}, [HCl] = 0.2 mM.

3.5.2 Heterogeneous population

The model file for XPPAUT is given in Appendix, model 2, for a population of vesicles with a distribution in enzyme loading. The vesicles were prepared in experiments with a hydrating solution with urease = 5.45 mg ml^{-1} = 220 U ml^{-1} (Mr = 545 kDa) and the mass of urease in a sample may be estimated as 40/600 × 100% = 6.7%, to give [E] = 0.7 μM (SI 1.2). Simulations were performed

assuming an encapsulation efficiency of 25%. The average concentration of enzyme, <E> = 55 U ml^{-1}, is therefore 0.175 μM in a volume of 1 μL. This suggests a value of 1.05 × 10^{11} enzyme molecules in 2.4 × 10^{11} vesicles, or an average of 0.44 molecules per vesicle. The probability of n molecules per vesicle $P(X=n)$ was calculated from a Poisson distribution with λ = 0.44 for X = 0 … 5. We note that earlier work has demonstrated that vesicle solute occupancy may follow a power law distribution, however here our main aim is to determine the influence of enzyme distribution on the collective dynamics so the form of the distribution was not further explored.[30] The vesicle volume fraction was estimated as $\phi = NVi/Vo = 2 \times 10^{-3}$, with total volume of vesicles = 1.0 μL. In order to simulate a population of 2.4 × 10^{11} nanovesicles, the number of enzyme molecules (X*P*2.4x10^{11}) in the corresponding volume (P*1 μL) was used to calculate the average enzyme concentration in U ml^{-1} (1 mol L^{-1} = 3.14 x 10^{8} U ml^{-1}) for that volume fraction. The rate of change of Ao was given by:

$$\frac{dAo}{dt} = g(A_o) + \emptyset\frac{3P}{r}\sum_{j=0}^{5} w_j(A_i - A_o) \qquad \text{(SE11)}$$

where w_j is the fraction of the vesicle volume with a given enzyme number/concentration.

3.6 Microvesicles

The XPPAUT file is given in Appendix, model 3. The initial conditions in the microvesicles were: [pyranine] = 50 μM, and [HA] = 2.5 – 7.5 mM (where HA = acetic acid) and outer solution: $[\text{urea}]_o$ = 80 mM; [HA] = 2.5 – 7.5 mM. The vesicle volume fraction was $\phi = NV_i/V_o = 0.018$.

In order to simulate a population of microvesicles with different enzyme amount and sizes for Figure 6, a bivariate histogram of X = 30 bins was taken with weighting factor $w_{j,k}$ for the fraction of vesicles in each bin:

$$\frac{dAo}{dt} = g(A_o) + \frac{4\pi P_i}{V_o}\sum_j\sum_k w_{j,k} r_{j,k}^2 (A_i - A_o) \qquad \text{(SE12)}$$

where $w_{j,k}$ was calculated from the dot product of a normal probability distribution for the enzyme in the vesicles and the experimental probability distribution for the vesicle sizes (Figure 6). The values of $w_{j,k}$ are included in the ode file. The enzyme concentration was varied from $X = 60 – 100$ U ml^{-1}, with mean = 80 and sd = 10. A normal distribution was used as in a sample volume of 114 vesicles, the volume was 1.9×10^{-4} μL and there were 2.6×10^{6} urease molecules with [E] = 0.25 μM and an average of 2.3×10^{5} molecules per vesicle. Again we note that large variations in intravesicle solute concentration have been reported, however our goal here was to observe the influence of heterogeneity on the collective dynamics, rather than quantitatively match experimental results, so we do not explore the nature of the distribution further here.[30]

The pH in time, vesicle diameter and corresponding clock time and initial and final pH are shown in Figure S11. The initial pH was influenced by both the enzyme content (yellow circles) and the diameter, with a smaller diameter and smaller enzyme concentration favouring low pH.

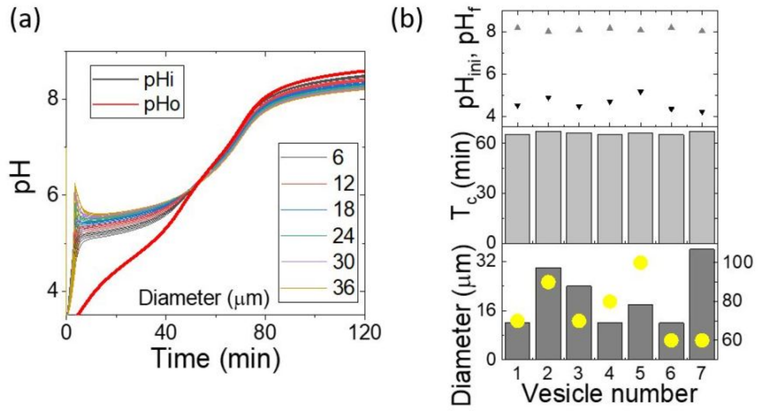

Figure S11. Simulations of heterogeneous microvesicles with [pyranine] = 50 μM, in a solution of [urea] = 32 mM and [acetic acid] = 7.5 mM and $\phi = NV_i/V_o = 0.018$. (a) pH in time and (b) Diameter (bars), enzyme concentration (yellow circles) and corresponding clock time and initial and final pH in seven of the vesicles.

The average T_c and initial and final pH for the population of vesicles with changes in the concentration of initial acid are shown in Figure S12(a) and (b).

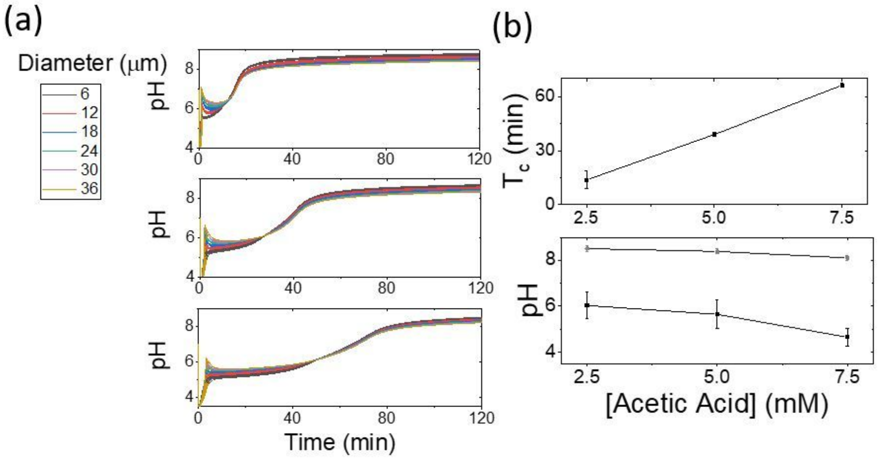

Figure S12. Simulations of heterogeneous microvesicles with [pyranine] = 50 μM, in a solution of [urea] = 32 mM and [acetic acid] = 2 - 7.5 mM and $\phi = NV_i/V_o = 0.018$. (a) Average internal pH in the vesicles (black) and pH in the outer solution (red) (b) average clock time and (c) initial and final pH as a function of initial acid concentration.

References

(1) Jones, S. J. Investigating Nonlinear Enzyme Kinetics as an Internal Control System for Nanoreactor Drug Release. University of Leeds, 2018. https://etheses.whiterose.ac.uk/22207/.

(2) Colletier, J.-P.; Chaize, B.; Winterhalter, M.; Fournier, D. Protein encapsulation in liposomes: efficiency depends on interactions between protein and phospholipid bilayer. *BMC Biotechnol.* **2002**, *2,* p 9.

(3) Krajewska, B. Ureases I. Functional, catalytic and kinetic properties: A review. *J. Mol. Catal. B: Enzym.* **2009**, *59,* pp 9-21.

(4) Krajewska, B.; Van Eldik, R.; Brindell, M. Temperature- and pressure-dependent stopped-flow kinetic studies of jack bean urease. Implications for the catalytic mechanism. *J. Biol. Inorg. Chem.* **2012**, *17,* pp 1123-1134.

(5) Blakeley, R. L.; Zerner, B. Jack bean urease: the first nickel enzyme. *J. Mol. Catal.* **1984**, *23,* pp 263-292.

(6) Madeira, V. M. C. Incorporation of urease into liposomes. *BBA - General Subjects* **1977**, *499,* pp 202-211.
(7) Kano, K.; Fendler, J. H. Pyranine as a sensitive pH probe for liposome interiors and surfaces. pH gradients across phospholipid vesicles. *BBA - Biomembranes* **1978**, *509,* pp 289-299.
(8) Clement, N. R.; Gould, J. M. Pyranine (8-Hydroxy-1,3,6-pyrenetrisulfonate) as a Probe of Internal Aqueous Hydrogen Ion Concentration in Phospholipid Vesicles. *Biochemistry* **1981**, *20,* pp 1534-1538.
(9) Bizzarri, R.; Arcangeli, C.; Arosio, D.; Ricci, F.; Faraci, P.; Cardarelli, F.; Beltram, F. Development of a novel GFP-based ratiometric excitation and emission pH indicator for intracellular studies. *Biophys. J.* **2006**, *90,* pp 3300-3314.
(10) Clayton, T. D.; Byrne, R. H. Spectrophotometric seawater pH measurements: total hydrogen ion concentration scale calibration of m-cresol purple and at-sea results. *Deep-Sea Research Part I* **1993**, *40,* pp 2115-2129.
(11) Kučerka, N.; Nieh, M. P.; Katsaras, J. Fluid phase lipid areas and bilayer thicknesses of commonly used phosphatidylcholines as a function of temperature. *Biochimica et Biophysica Acta - Biomembranes* **2011**, *1808,* pp 2761-2771.
(12) Pautot, S.; Frisken, B. J.; Weitz, D. A. Production of unilamellar vesicles using an inverted emulsion. *Langmuir* **2003**, *19,* pp 2870-2879.
(13) Walde, P.; Cosentino, K.; Engel, H.; Stano, P. Giant Vesicles: Preparations and Applications. *ChemBioChem* **2010**, *11,* pp 848-865.
(14) Mobley, H. L. T.; Hausinger, R. P. Microbial ureases: Significance, regulation, and molecular characterization. *Microbiological Reviews* **1989**, *53,* pp 85-108.
(15) Frank, M. J. W.; Kuipers, J. A. M.; Van Swaaij, W. P. M. Diffusion coefficients and viscosities of CO2 + H2O, CO2 + CH3OH, NH3 + H2O, and NH3 + CH3OH liquid mixtures. *J. Chem. Eng. Data* **1996**, *41,* pp 297-302.
(16) Wrobel, M. M.; Bánsági Jr, T.; Scott, S. K.; Taylor, A. F.; Bounds, C. O.; Carranzo, A.; Pojman, J. A. PH wave-front propagation in the urea-urease reaction. *Biophys. J.* **2012**, *103,* pp 610-615.
(17) Krajewska, B.; Ciurli, S. Jack bean (Canavalia ensiformis) urease. Probing acid–base groups of the active site by pH variation. *Plant Physiol. Biochem.* **2005**, *43,* pp 651-658.
(18) Dixon, N. E.; Riddles, P. W.; Gazzola, C.; Blakeley, R. L.; Zerner, B. Jack bean urease (EC 3.5.1.5). V. On the mechanism of action of urease on urea, formamide, acetamide, N-methylurea, and related compounds. *Can. J. Biochem.* **1980**, *58,* pp 1335-1344.
(19) Lopreore, C.; Byers, L. D. The urease-catalyzed hydrolysis of thiourea and thioacetamide. *Arch. Biochem. Biophys.* **1998**, *349,* pp 299-303.

(20) Hu, G.; Pojman, J. A.; Scott, S. K.; Wrobel, M. M.; Taylor, A. F. Base-catalyzed feedback in the urea-urease reaction. *J. Phys. Chem. B* **2010**, *114,* pp 14059-14063.
(21) Wang, X.; Conway, W.; Burns, R.; McCann, N.; Maeder, M. Comprehensive study of the hydration and dehydration reactions of carbon dioxide in aqueous solution. *J. Phys. Chem. A* **2010**, *114,* pp 1734-1740.
(22) Avnir, Y.; Barenholz, Y. pH determination by pyranine: Medium-related artifacts and their correction. *Anal. Biochem.* **2005**, *347,* pp 34-41.
(23) Zwolinski, B. J.; Eyring, H.; Reese, C. E. Diffusion and membrane permeability. I. *Journal of Physical & Colloid Chemistry* **1949**, *53,* pp 1426-1453.
(24) Missner, A.; Pohl, P. 110 years of the Meyer-Overton rule: Predicting membrane permeability of gases and other small compounds. *ChemPhysChem* **2009**, *10,* pp 1405-1414.
(25) Paula, S.; Volkov, A. G.; Van Hoek, A. N.; Haines, T. H.; Deamer, D. W. Permeation of protons, potassium ions, and small polar molecules through phospholipid bilayers as a function of membrane thickness. *Biophys. J.* **1996**, *70,* pp 339-348.
(26) Lande, M. B.; Donovan, J. M.; Zeidel, M. L. The relationship between membrane fluidity and permeabilities to water, solutes, ammonia, and protons. *J. Gen. Physiol.* **1995**, *106,* pp 67-84.
(27) Xiang, T. X.; Anderson, B. D. Permeability of acetic acid across gel and liquid-crystalline lipid bilayers conforms to free-surface-area theory. *Biophys. J.* **1997**, *72,* pp 223-237.
(28) Decoursey, T. E. Voltage-gated proton channels and other proton transfer pathways. *Physiol. Rev.* **2003**, *83,* pp 475-579.
(29) Ermentrout, B. *Simulating, Analyzing, and Animating Dynamical Systems: A Guide to XPPAUT for Researchers and Students* SIAM, 2002.

(30) Stano, P.; de Souza, T. P.; Carrara, P.; Altamura, E.; D'Aguanno, E.; Caputo, M.; Luisi, P. L.; Mavelli, F. Recent Biophysical Issues About the Preparation of Solute-Filled Lipid Vesicles. *Mechanics of Advanced Materials and Structures* **2015**, *22,* pp 748-759.

Appendix 1: Ode files for simulations in XPPaut

Model 1

```
#urea-urease reaction in identical nanovesicles: rate equations
#vesicles
U'=-k1*E*U/((KM+U*(1+U/KS))*(1+Kes2/H+H/Kes1)*(1+NH4/Kp))+l*Pu/r*(Uo-U)
NH3'=2*k1*E*U/((KM+U*(1+U/KS))*(1+Kes2/H+H/Kes1)*(1+NH4/Kp))+k2*NH4-
k2r*NH3*H+l*Pn/r*(NH3o-NH3)
NH4'=-k2*NH4+k2r*NH3*H
CO2'=k1*E*U/((KM+U*(1+U/KS))*(1+Kes2/H+H/Kes1)*(1+NH4/Kp))-
k3*CO2+k3r*H*HCO3+l*Pc/r*(CO2o-CO2)
```

```
HCO3'=k3*CO2-k3r*HCO3*H-k4*HCO3+k4r*CO3*H
CO3'=k4*HCO3-k4r*CO3*H
H'=k2*NH4-k2r*NH3*H+k4*HCO3-k4r*CO3*H+k5-k5r*H*OH+k3*CO2-k3r*HCO3*H+k6*pyOH-
k6r*H*pyO+l*Php/r*(Ho-H)
OH'=k5-k5r*H*OH
pyOH'=-k6*pyOH+k6r*H*pyO
pyO'=k6*pyOH-k6r*H*pyO

#outer solution
Uo'=-(3*Pu/r)*f*(Uo-U)
NH3o'=k2*NH4o-k2r*NH3o*Ho-(l*Pn/r)*f*(NH3o-NH3)
NH4o'=-k2*NH4o+k2r*NH3o*Ho
CO2o'=-k3*CO2o+k3r*Ho*HCO3o-(l*Pc/r)*f*(CO2o-CO2)
HCO3o'=k3*CO2o-k3r*HCO3o*Ho-k4*HCO3o+k4r*CO3o*Ho
CO3o'=k4*HCO3o-k4r*CO3o*Ho
Ho'=k2*NH4o-k2r*NH3o*Ho+k4*HCO3o-k4r*CO3o*Ho+k5-k5r*Ho*OHo+k3*CO2o-
k3r*HCO3o*Ho+k6*pyOHo-k6r*Ho*pyOo-(l*Php/r)*f*(Ho-H)
OHo'=k5-k5r*Ho*OHo
pyOHo'=-k6*pyOHo+k6r*Ho*pyOo
pyOo'=k6*pyOHo-k6r*Ho*pyOo

aux pH=-log(H)/log(10)
aux pho = -log(Ho)/log(10)

#parameters  E = u/ml and P in m min-1, rate constants min-1
par l=3.0,k1=0.00022,E=55,r=100e-9,f=0.002
par Pu=6e-7,Pn=6e-3,Pc=6e-5,Php=0
par k7=4.7e7,k7r=2.7e12
par KM=0.003,KS=3,KP=0.02,Kes1=5e-6,Kes2=2e-9
par k2=1440,k2r=2.58e12,k3=2.22,k3r=4740000,k4=168,k4r=3e12, k5=0.06, k5r=6e12,
k6=60,k6r=1.5e9

#initial conditions
init U=0.00,NH3=0,NH4=0,CO2=0,HCO3=0,CO3=0,H=2e-4,OH=1e-10,pyOH=0.005
init Uo=0.05,NH3o=0,NH4o=0,CO2o=0,HCO3o=0,CO3o=0,Ho=2e-4,OHo=1e-
10,pyOHo=0.00,pyOo=0.00
#numerical stuff
@ total=90,dt=0.1,tol=1e-7, meth=stiff,bandup=13,bandlo=13, bounds=10000
@ xplot=t,yplot=pH,xhi=90,ylo=1,yhi=14
Done
```

Model 2. Nanovesicle heterogeneous population

```
#urea-urease reaction in nanovesicles with a distribution of enzyme: rate equations
#vesicles
f(e,u,h,nh)=k1*e*u/((KM+u*(1+u/KS))*(1+Kes2/h+h/Kes1)*(1+a*nh/Kp))
f2(nh,n,h)=k2*nh-k2r*n*h
f3(c,h,hco)=k3*c-k3r*h*hco
f4(hco,co,h)=k4*hco-k4r*co*h
f5(h,oh)=k5-k5r*h*oh
f6(pyh,py,h)=k6*pyH-k6r*py*h
```

```
%[1..6]
e[j]'=0
w[j]'=0
u[j]'=-f(e[j],u[j],h[j],nh[j])-l*pu/r*(u[j]-u7)
n[j]'=2*f(e[j],u[j],h[j],nh[j])+f2(nh[j],n[j],h[j])-l*pn/r*(n[j]-n7)
nh[j]'=-f2(nh[j],n[j],h[j])
c[j]'=f(e[j],u[j],h[j],nh[j])-f3(c[j],h[j],hco[j])-l*pc/r*(c[j]-c7)
hco[j]'=f3(c[j],h[j],hco[j])-f4(hco[j],co[j],h[j])
co[j]'=f4(hco[j],co[j],h[j])
h[j]'=f2(nh[j],n[j],h[j])+f3(c[j],h[j],hco[j])+f4(hco[j],co[j],h[j])+f5(h[j],oh[j])+f6(pyh[j],h[j],py[j])-
l*php/r*(h[j]-h7)
oh[j]'=f5(h[j],oh[j])
pyh[j]'=-f6(pyh[j],py[j],h[j])
py[j]'=f6(pyh[j],py[j],h[j])
%

#surrounding solution
u7'=-(l*pu/r)*fv*(sum(0,5)of(shift(w1,12*i')*(u7-shift(u1,12*i'))))
n7'=f2(nh7,n7,h7)-(l*pn/r)*fv*(sum(0,5)of(shift(w1,12*i')*(n7-shift(n1,12*i'))))
nh7'=-f2(nh7,n7,h7)
c7'=-f3(c7,h7,hco7)-(l*pc/r)*fv*(sum(0,5)of(shift(w1,12*i')*(c7-shift(c1,12*i'))))
hco7'=f3(c7,h7,hco7)-f4(hco7,co7,h7)
co7'=f4(hco7,co7,h7)
h7'=f2(nh7,n7,h7)+f3(c7,h7,hco7)+f4(hco7,co7,h7)+f5(h7,oh7)+f6(pyh7,py7,h7)-
(l*php/r)*fv*(sum(0,5)of(shift(w1,12*i')*(h7-shift(h1,12*i'))))
oh7'=f5(h7,oh7)
pyh7'=-f6(pyh7,py7,h7)
py7'=f6(pyh7,py7,h7)

aux pH[1..6]=-log(h[j])/log(10)
aux pH7 = -log(h7)/log(10)

#parameters  E = u/ml and P in m min-1, rate constants min-1
par l=3.0,a=1,b=1,k1=0.00022,r=100e-9,fv=0.002
par pu=6e-7,pn=6e-3,pc=6e-5,php=0
par k7=4.7e7,k7r=2.7e12
par KM=0.003,KS=3,KP=0.02,Kes1=5e-6,Kes2=2e-9
par k2=1440,k2r=2.58e12,k3=2.22,k3r=4740000,k4=168,k4r=3e12, k5=0.06, k5r=6e12,
k6=60,k6r=1.5e9

#initial conditions
init e1=0
init e2=124
init e3=248
init e4=372
init e5=496
init e6=621
init w1=0.644,w2=0.283,w3=0.06234,w4=0.00914,w5=0.00101,w6=8.851e-5
init U[1..6]=0.00
```

```
init H[1..6]=2e-4
init OH[1..6]=1e-10
init pyH[1..6]=0.005
init U7=0.05
init H7=2e-4
init OH7=1e-10
init pyH7=0.00

#numerical stuff
@ total=90,dt=0.1,tol=1e-7, meth=stiff,bandup=12,bandlo=12, bounds=10000
@ xplot=t,yplot=pH1,xhi=90,ylo=1,yhi=14
Done
```

Model 3. Microvesicle heterogeneous population

```
#urea-urease reaction in vesicles- r is radius in m
#vesicles
f(e,u,h,nh)=k1*e*u/((KM+u*(1+b*u/KS))*(1+c*Kes2/h+c*h/Kes1)*(1+b*nh/Kp))
f2(nh,n,h)=k2*nh-k2r*n*h
f3(c,h,hco)=k3*c-k3r*h*hco
f4(hco,co,h)=k4*hco-k4r*co*h
f5(h,oh)=k5-k5r*h*oh
f6(pyh,py,h)=k6*pyH-k6r*py*h
f7(ah,h,a)=k7*ah-k7r*h*a

%[1..30]
e[j]'=0
r[j]'=0
w[j]'=0
u[j]'=-f(e[j],u[j],h[j],nh[j])-l*pu/r[j]*(u[j]-u31)
n[j]'=2*f(e[j],u[j],h[j],nh[j])+f2(nh[j],n[j],h[j])-l*pn/r[j]*(n[j]-n31)
nh[j]'=-f2(nh[j],n[j],h[j])
c[j]'=f(e[j],u[j],h[j],nh[j])-f3(c[j],h[j],hco[j])-l*pc/r[j]*(c[j]-c31)
hco[j]'=f3(c[j],h[j],hco[j])-f4(hco[j],co[j],h[j])
co[j]'=f4(hco[j],co[j],h[j])
h[j]'=f2(nh[j],n[j],h[j])+f3(c[j],h[j],hco[j])+f4(hco[j],co[j],h[j])+f5(h[j],oh[j])+f6(pyh[j],h[j],py[j])+f7(ah[j]
,h[j],a[j])-l*php/r[j]*(h[j]-h31)
oh[j]'=f5(h[j],oh[j])
pyh[j]'=-f6(pyh[j],py[j],h[j])
py[j]'=f6(pyh[j],py[j],h[j])
ah[j]'=-f7(ah[j],h[j],a[j])-l*pah/r[j]*(ah[j]-ah31)
a[j]'=f7(ah[j],h[j],a[j])
%

#surrounding solution
u31'=-(4.0*pi*pu*Nd)*(sum(0,29)of(shift(w1,15*i')*shift(r1,15*i')*shift(r1,15*i')*(u31-
shift(u1,15*i'))))
n31'=f2(nh31,n31,h31)-
(4.0*pi*pn*Nd)*(sum(0,29)of(shift(w1,15*i')*shift(r1,15*i')*shift(r1,15*i')*(n31-shift(n1,15*i'))))
nh31'=-f2(nh31,n31,h31)
```

```
c31'=-f3(c31,h31,hco31)-
(4.0*pi*pc*Nd)*(sum(0,29)of(shift(w1,15*i')*shift(r1,15*i')*shift(r1,15*i')*(c31-shift(c1,15*i'))))
hco31'=f3(c31,h31,hco31)-f4(hco31,co31,h31)
co31'=f4(hco31,co31,h31)
h31'=f2(nh31,n31,h31)+f3(c31,h31,hco31)+f4(hco31,co31,h31)+f5(h31,oh31)+f6(pyh31,py31,h31)+f
7(ah31,h31,a31)-(4.0*pi*php*Nd)*(sum(0,29)of(shift(w1,15*i')*shift(r1,15*i')*shift(r1,15*i')*(h31-
shift(h1,15*i'))))
oh31'=f5(h31,oh31)
pyh31'=-f6(pyh31,py31,h31)
py31'=f6(pyh31,py31,h31)
ah31'=-f7(ah31,h31,a31)-
(4.0*pi*pah*Nd)*(sum(0,29)of(shift(w1,15*i')*shift(r1,15*i')*shift(r1,15*i')*(ah31-shift(ah1,15*i'))))
a31'=f7(ah31,h31,a31)

aux pH[1..30]=-log(h[j])/log(10)
aux ph31 = -log(h31)/log(10)

#parameters  - rate constants are in min-1, P in m/min, rate constants min-1
par l=3.0,b=1,c=1,k1=0.00022,Nd=7.1E12
par pu=6e-7,pn=6e-3,pc=6e-5,php=0,pah=6e-6
par KM=0.003,KS=3,KP=0.02,Kes1=5e-6,Kes2=2e-9
par
k2=1440,k2r=2.58e12,k3=2.22,k3r=4740000,k4=168,k4r=3e12,k5=0.06,k5r=6e12,k6=60,k6r=1.5e9
par k7=4.7e7,k7r=2.7e12

#initial conditions in M, e = u/ml, d in m
init e[1..6]=60
init e[7..12]=70
init e[13..18]=80
init e[19..24]=90
init e[25..30]=100
init r1=3e-6,r7=3e-6,r13=3e-6,r19=3e-6,r25=3e-6
init r2=6e-6,r8=6e-6,r14=6e-6,r20=6e-6,r26=6e-6
init r3=9e-6,r9=9e-6,r15=9e-6,r21=9e-6,r27=9e-6
init r4=12e-6,r10=12e-6,r16=12e-6,r22=12e-6,r28=12e-6
init r5=15e-6,r11=15e-6,r17=15e-6,r23=15e-6,r29=15e-6
init r6=18e-6,r12=18e-6,r18=18e-6,r24=18e-6,r30=18e-6
init w1=0.0074956,w2=0.018589,w3=0.011522,w4=0.0036408,w5=0.0017561,w6=0.00051404
init w7=0.044974,w8=0.11153,w9=0.069132,w10=0.021845,w11=0.010537,w12=0.0030842
init w13=0.070459,w14=0.17474,w15=0.10831,w16=0.034223,w17=0.016508,w18=0.0048319
init w19=0.037478,w20=0.092945,w21=0.05761,w22=0.018204,w23=0.0087807,w24=0.0025702
init w25=0.010494,w26=0.026025,w27=0.016131,w28=0.0050971,w29=0.0024586,w30=0.00071965

init h[1..30]=1e-7
init oh[1..30]=1e-7
init pyh[1..30]=50e-6
init ah[1..30]=0.0075
init u[1..30]=0.08
init u31=0.08,h31=1e-7,oh31=1e-7,ah31=0.0075
```

```
#numerical stuff
@ total=120,dt=0.05,tol=1e-6, meth=stiff,bandup=15,bandlo=15, bounds=10000
@ xplot=t,yplot=pH1,xhi=90,ylo=3,yhi=10
Done
```
